# Identification of Candidate Genes Responsible for Flower Colour Intensity in *Gentiana triflora*

**DOI:** 10.3389/fpls.2022.906879

**Published:** 2022-06-22

**Authors:** Keisuke Tasaki, Aiko Watanabe, Keiichirou Nemoto, Shigekazu Takahashi, Fumina Goto, Nobuhiro Sasaki, Takashi Hikage, Masahiro Nishihara

**Affiliations:** ^1^Iwate Biotechnology Research Center, Kitakami, Japan; ^2^Hachimantai City Floricultural Research and Development Center, Hachimantai, Japan

**Keywords:** anthocyanin, DNA marker, flower colour intensity, gentian, MIF1, RNA-seq

## Abstract

Gentians cultivated in Japan (*Gentiana triflora* and *Gentiana scabra* and hybrids) have blue flowers, but flower colour intensity differs among cultivars. The molecular mechanism underlying the variation in flower colour intensity is unclear. Here, we produced F_2_ progeny derived from an F_1_ cross of intense- and faint-blue lines and attempted to identify the genes responsible for flower colour intensity using RNA-sequencing analyses. Comparative analysis of flower colour intensity and transcriptome data revealed differentially expressed genes (DEGs), although known flavonoid biosynthesis-related genes showed similar expression patterns. From quantitative RT-PCR (qRT-PCR) analysis, we identified two and four genes with significantly different expression levels in the intense- and faint-blue flower lines, respectively. We conducted further analyses on one of the DEGs, termed *GtMIF1*, which encodes a putative mini zinc-finger protein homolog, which was most differently expressed in faint-blue individuals. Functional analysis of *GtMIF1* was performed by producing stable tobacco transformants. *GtMIF1*-overexpressing tobacco plants showed reduced flower colour intensity compared with untransformed control plants. DNA-marker analysis also confirmed that the *GtMIF1* allele of the faint-blue flower line correlated well with faint flower colour in F_2_ progeny. These results suggest that *GtMIF1* is one of the key genes involved in determining the flower colour intensity of gentian.

## Introduction

Flower colour is one of the most important traits in ornamental plants. Consumers prefer a variety of flower colours; therefore, breeders are continually striving to produce novel varieties with different colours. In most cases, new varieties are produced by conventional cross-breeding and repeated selections, but several techniques, such as mutation, transgenes, and molecular markers, are also useful (Yi Li, 2006; Nishihara and Nakatsuka, 2010; Singh, 2014; Zhao and Tao, 2015; Noman et al., 2017). Typical biotechnological products are transgenic roses and carnations with a modified bluish flower colour, which does not exist in nature. These varieties have been commercially available for over a decade. Development of blue chrysanthemum has also been achieved by genetic engineering (Noda, 2018), although commercial use has not yet been approved owing to government GMO regulations. More recently, genome editing technologies have been developed, including the CRIPSR/Cas9 system, which has been used to alter flower colour in several plant species, including morning glory (Watanabe et al., 2017, 2018), torenia (Nishihara et al., 2018a), and petunia (Yu et al., 2021).

Colour intensity is another aspect of flower colour. Even if the same pigments accumulate in flower petals, the amounts of accumulated pigment greatly affect the flower colour visible to the human eye. Faint colours are preferable in some cases, while intense colour may be preferred in others. Intermediate colour may also be required in some situations. Therefore, breeding efforts have focused on flower colour intensity and cultivars with varying degrees of flower colour intensity are produced for most ornamental plants. Scientists have been trying to elucidate the mechanisms of flower colour variations for many years. The main flower pigments are flavonoids, carotenoids, and betalains (Tanaka et al., 2008; Ohmiya, 2011; Polturak and Aharoni, 2018). The former two are widely distributed in the plant kingdom and responsible for not only flower colour but also for the colour of various organs, including the leaves, stems, roots, and fruits. Betalains are restricted to the order Caryophyllales and are responsible for colouration of various organs (Polturak and Aharoni, 2018). Each biosynthetic pathway has been well characterised, and most genes involved in the biosynthesis of these pigments have already been identified.

In Japan, cultivated gentians (*Gentiana triflora* and *Gentiana scabra* and their hybrids; Japanese gentian from hereon) have been used extensively as cut flowers and potted plants for ornamental purposes for more than half a century (Kodama, 2006; Nishihara et al., 2018b). The wild-type flower colour of *Gentiana* species is blue, and the main pigment responsible for that colour is a delphinidin-based polyacylated anthocyanin, gentiodelphin (Goto et al., 1982). Using natural flower colour mutants as breeding materials, pink- and white-flowered cultivars have been produced (Nishihara et al., 2018b). A pink-flowered gentian has also been produced using ion beam irradiation (Sasaki et al., 2018), and red-flowered gentian cultivars have been produced using the embryo rescue technique (Eason et al., 2007). Recently, we clarified that the co-pigmentation effects of novel xanthones on cyanidin 3-*O*-glucoside contributed to vivid red flower colour (Sasaki et al., 2021). Colour patterns can also enhance the ornamental value of flowers. In gentian, we recently revealed that a striped bicolour phenotype resulted from post-transcriptional gene silencing of the chalcone synthase gene (*CHS*; Ohta et al., 2021). Chlorophyll also contributes to formation of the green colour of ornamental flowers; uniquely, gentian flowers have green spots consisting of functional chloroplasts in restricted areas of epidermal cells (Takahashi et al., 2020).

Among these flower colour variations, blue is the most popular and comprises more than 80% of gentian production in Iwate prefecture, Japan. The blue colour varies from faint purple to dark blue, depending on the cultivar. Most flavonoid biosynthetic genes and their regulatory genes have been identified in gentian (Nishihara et al., 2018b). Using genome-editing technology targeting anthocyanin-modifying enzymes, such as glucosyltransferase and acyltransferase, we confirmed that these enzymes were indispensable for gentiodelphin production and contributed to the deep, vivid blue flower colour *in planta* (Tasaki et al., 2019). More recently, we also identified a glutathione *S*-transferase (*GST1*) gene involved in transporting anthocyanin into the vacuole in Japanese gentian (Tasaki et al., 2020). The accumulated anthocyanin is the main factor determining flower colour in gentian.

Flower colour is affected not only by pigment quality (the types of accumulated pigments), but also pigment quantity (the amounts of accumulated pigments). Several studies have attempted to elucidate the regulatory mechanism(s) of flower colour intensity in floricultural plants. For example, Ohno et al. (2013) revealed that a basic helix–loop–helix transcription factor, DvIVS, determines flower colour intensity in cyanic dahlia cultivars. In *Cymbidium* orchids, the diversity of flower colour intensities and patterning is derived from subtle variations in the concentration and pattern of pigment accumulation *via* temporal and spatial regulation of anthocyanin biosynthesis (Wang et al., 2014). Transcriptome analysis of *Paeonia ostia* suggested that differential expression of genes encoding anthocyanin repressors was the main factor responsible for flower colour intensity (Gao et al., 2015). However, there are no scientific studies of flower colour intensity in Japanese gentian, and the mechanism(s) regulating intensity is unknown. Thus, in the present study we attempted to identify the genes responsible for flower colour intensity in Japanese gentian. For this purpose, F_2_ progenies, derived by crossing F_1_ lines with intense- and faint-blue flowers, were subjected to transcriptome analysis using RNA-sequencing. Of the differentially expressed genes (DEGs) between intense and faint-blue flowers, we focused on a putative mini zinc-finger (MIF) protein homolog, termed *GtMIF1*, which showed the greatest difference in expression between faint and intense-blue flowers and performed functional analysis using stable tobacco transformants. We also developed a DNA marker and performed validity tests using F_2_ plants. The results suggest that *GtMIF1* is involved in the regulation of flower colour intensity in Japanese gentian. Furthermore, the novel biological function of *GtMIF1* would contribute in gaining insight into the functional roles of this protein family in higher plants.

## Materials and Methods

### Plant Materials

**Figure 1:**
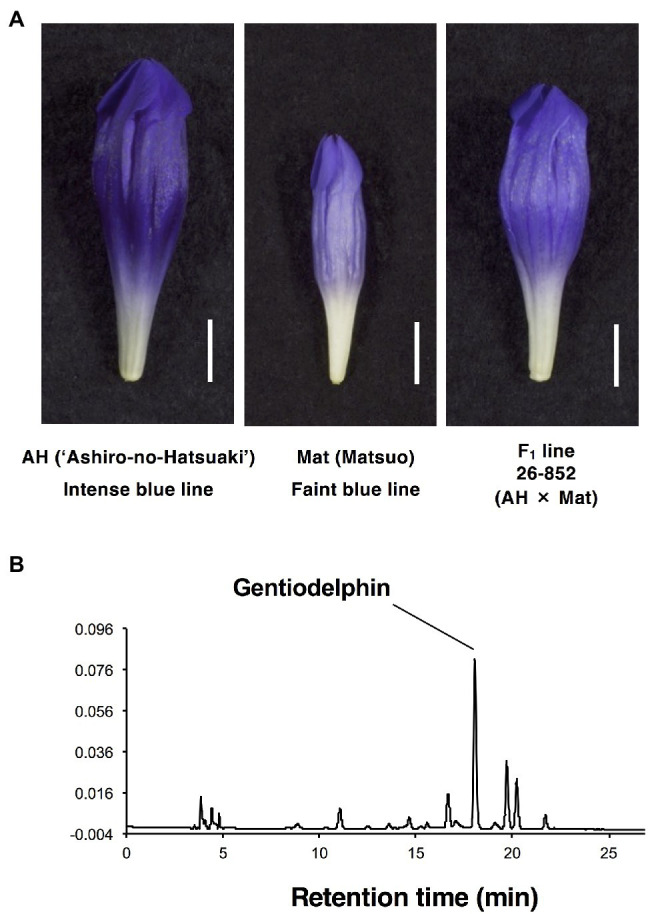
Flower phenotypes of parents and F_1_ used for crossing in this study. **(A)** AH, intense-blue doubled haploid line 26-853_Go1230 in gentian cultivar ‘Ashiro-no-Hatsuaoi’. Mat, faint-blue flower line 24-2007MAR7-Go5 doubled haploid. F_1_ line 26-852 (AH x Mat). **(B)** Chromatogram of anthocyanin pigment extracts from petals of the AH line at 520 nm.

In this study, we used *G. triflora* double haploid (DH) lines produced by unfertilised ovule culture (Doi et al., 2013). For development of progeny segregated by flower colour intensity, ‘Ashiro-no-Hatsuaki’-derived DH line (AH), with intense-blue flowers, and ‘Matsuo’-derived DH line (Mat), with faint flowers, were used to produce F_1_ line (26-852) (Figure 1). The F_2_ progeny line (27-867) was produced by self-pollination of one F_1_ progeny line (26-852-20). All seedlings were grown in cell trays at Hachimantai City Floricultural Research and Development Centre and moved to Iwate Biotechnology Research Centre for flowering. Transplanted potted F_2_ seedlings were cultivated in a closed glass greenhouse maintained at 22°C–27°C under natural sunlight with supplementary lighting with halogen lamps for forcible induction of flowering in the first year. For production of reliable reference sequences, the ‘Iwate-Otome’-derived DH line (IO) and F_1_ line 26-850 (AH × IO) were also used for *de novo* assembly.

### Analysis of Flower Petal Colour Intensity

For evaluation of flower colour intensity, the colorimetric value L^*^, which indicates the lightness of the adaxial surface of the limb area of fresh petals at anthesis, was measured using a CM-3600A spectrophotometer (Konica Minolta, Tokyo, Japan) as described previously (Tasaki et al., 2019).

### Analysis of Accumulated Pigments in Flower Petals

The anthocyanin composition and content of petal extracts from parents, F_1_ and F_2_ progeny were analysed using high-performance liquid chromatography (HPLC). Fresh petals from the first opened flower of each plant were collected and stored at −80°C until use. The cryopreserved petals were immediately soaked in 80% methanol containing 0.1% (v/v) trifluoroacetic acid for 24 h to extract the anthocyanins, filtered using a spin column, and then separated with an HPLC–photodiode array detector system (L-2455 Diode Array Detector, AS-4010 Auto sampler and D-2000 Elite chromatography DATA station, Hitachi High-Technologies, Tokyo, Japan; LC-20AT pump, Shimadzu Co., Kyoto, Japan) equipped with an ODS column (inner diameter 4.6 × 250 mm; Wakopak Handy ODS, Wako Pure Chemical Industries, Ltd., Osaka, Japan) using a linear gradient elution (1 ml min^−1^) of 10%–40% acetonitrile in 1.0% aqueous phosphoric acid for 30 min.

### Selection of DEGs Between Intense- and Faint-Blue Gentian Lines by RNA-Sequencing

Total RNAs were extracted from the petals and leaves using an RNeasy Plant Mini Kit (Qiagen, Hilden, Germany). RNA-sequencing (RNA-seq) steps, mRNA purification from the total RNA, library construction, and Illumina paired-end sequencing on the NextSeq 500 system (150 bp, paired-end; Illumina Inc., San Diego, CA, United States) are summarised in Supplementary Table S1. All RNA-seq libraries were constructed independently from each RNA extract of the plant tissues. Raw sequence reads were pre-processed using a FASTX toolkit. *De novo* assembly for development of a reference for transcriptome analysis was performed using Trinity (v2.1.1) with parameter ‘--min_contig_length 500’. This reference was constructed using preprocessed sequence reads of petals in floral development stages S1–S4 of the *G. triflora* lines, AH, Mat, and F_1_ line 26-852, ‘Iwate-Otome’ DH (IO), AH × IO F_1_ line 26-850, and that of fresh leaf of AH and Mat lines. Obtained contigs were annotated using blastx v2.2.26 (uniprot_sprot) and blastn v2.2.26 (nr) on Maser (Management and Analysis System for Enormous Reads; Kinjo et al., 2018). The summary of the results of *de novo* assembly and annotation is shown in Supplementary Table S2.

Two types of expression profiles for DEG analysis were constructed using RNA-seq reads of the petals of the first one just-opened flower of five F_2_ intense-blue lines (27-867-048, 27-867-060, 27-867-030, 27-867-003, and 27-867-067) and five F_2_ faint-blue lines (27-867-056, 27-867-077, 27-867-008, 27-867-087, and 27-867-076), and the petals of floral development stages S1–S4 of AH and Mat, respectively. FPKM values among these samples were estimated using RSEM software (v1.5.3; Li and Dewey, 2011), with the reference constructed by *de novo* assembly consisting of 148,061 contigs. To identify DEGs between the two F_2_ groups (intense- and faint-blue flower lines), a Trimmed mean of M-values (TMM) software (Robinson et al., 2010; Robinson and Oshlack, 2010) was first used to normalise the FPKM values among the F_2_ samples; then, NOISeq software (Tarazona et al., 2011, 2015) was used with the expression profiles of five individual plants from each F_2_ intense- and faint-blue lines as biological replicates. In this analysis, 7,110 annotated contigs with high expression levels were used as the target genes from 148,061 contigs. Normalisation of the FPKM values of the petals of S1–S4 of AH and Mat was also performed using the TMM software.

### Quantitative RT-PCR Analysis of Gentian

Total RNAs were extracted from representative F_2_ individuals of each intense- and faint-blue flower line. Quantitative RT-PCR (qRT-PCR) was performed using the QuantStudio 3 Real-Time PCR system, as described previously (Sasaki et al., 2021). Relative expression levels of two IBGs and four FBGs were determined. *GtUBQ* was used as an internal standard.

### Cloning of *GtMIF1*

To isolate *GtMIF1-1* and *GtMIF1-2*, total RNAs were extracted from flowers of F_2_ line plants that showed lower L^*^ values, using an RNeasy Plant Mini Kit (Qiagen). For synthesising first-strand cDNA, 100 ng of total RNA was reverse transcribed using the ReverTra Ace qPCR RT Master Mix with gDNA Remover kit (Toyobo Co., Ltd., Osaka, Japan) according to the manufacturer’s instructions. The open reading frame (ORF) of *GtMIF1-1* and *GtMIF1-2* was amplified by PCR using PrimSTAR max DNA Polymerase (Takara Bio Inc., Shiga, Japan) and synthesised cDNA as a template. The DNA fragments were cloned into pDONR221, and the sequences were confirmed. Primer sets for cloning are shown in Supplementary Table S3.

### Production and Phenotype Analysis of Tobacco Transformants Overexpressing *GtMIF1*

To overexpress *GtMIF1*, cDNA containing an ORF was amplified using primers, GtMIF1Start (*Xba* I) and GtMIF1Stop (*Sac* I) using TaKaRa PrimeSTAR GXL DNA Polymerase and was subcloned into PCR-BluntII TOPO (Invitrogen, Carlsbad, CA, United States). The sequence was confirmed using the ABI PRISM 3500 Genetic Analyser (Applied Biosystems, Foster City, CA, United States). The *GtMIF1* fragment was cut out from the cloning vector and ligated into the XbaI and SacI sites of a binary vector, pSKAN35SGUS (Nakatsuka et al., 2012) to replace *GtMIF1* with *GUS*. To enhance expression, *Arabidopsis* ADH enhancer and terminator were inserted, resulting in pSKAN35SGtMIF1. The binary vector was transformed into *Agrobacterium tumefaciens* strain EHA101 by electroporation. Tobacco transformation and selection of T_1_ plants were performed as follows. Seeds of *Nicotiana tabacum* cv. SR1 were aseptically sown and grown for approximately 1 month on solidified Murashige and Skoog (MS) medium supplemented with 3% sucrose in a plastic box. Leaf sections were prepared and inoculated overnight with bacterial solution and co-cultivated for 2 days. Leaf sections were transferred to regeneration medium (solidified MS medium supplemented with 3% sucrose, 1 mg/L BAP, and 0.1 mg/L NAA) containing 100 mg/L kanamycin and 300 mg/L carbenicillin. Kanamycin-resistant shoots were excised from leaf sections and transferred to rooting medium (solidified MS medium supplemented with 3% sucrose) containing 100 mg/L kanamycin and 300 mg/L carbenicillin. Rooted plants were acclimatised and grown in a closed greenhouse until flowering. T_1_ seeds were produced by selfing of each T_0_ plant. Obtained T_1_ seeds were sown on MS medium supplemented with 3% sucrose and 100 mg/L kanamycin. Kanamycin-resistant seedlings were acclimatised, and plant phenotypes were observed in a closed greenhouse.

### Quantitative RT-PCR Analysis of Tobacco Plants

Total RNAs were extracted using an RNeasy Plant Mini Kit (Qiagen) from flowers of three T_1_ lines and the untransformed control, SR1. They were subjected to qRT-PCR analysis as described above. Introduced foreign *GtMIF1* and endogenous gene expression levels, including *NtCHS*, *NtFLS*, *NtANS* and transcription factors, *NtAn1a*, *NtAn1b* and *NtAn2*, were calibrated using *UBQ* gene expression as an internal standard. A minimum of five flowers were used for this analysis. All primers used are listed in Supplementary Table S3.

### Development of Molecular DNA Markers for *GtMIF1*

To assess whether *MIF1* inheritance and flower colour intensity were correlated, PCR-based markers to distinguish the presence of the *GtMIF1* allele in faint-blue flower lines were applied to the F_2_ progeny. Because the F_2_ individuals used for the RNA-seq analysis died during the experiment, we sowed new seeds of F_2_ progeny from the same plant of F_1_ line (26-852-20) and analysed their flower colour intensity by spectrophotometer for 2 years. DNA was extracted using a GenElute plant genomic DNA miniprep kit (Sigma-Aldrich, St. Louis, MO, United States) from leaves of each F_2_ progeny and subjected to PCR analysis. *GtMIF1* specifically amplifying the faint-blue *MIF1* allele was designed from the gentian draft genome (unpublished) and used for this analysis. Primer sets are shown in Supplementary Table S3. PCR was performed using MightyAmp DNA Polymerase Ver.3 (Takara Bio) according to the manufacturer’s instructions. Analysis of PCR products was performed using 1.6% agarose gel electrophoresis.

## Results

### Inheritance of Flower Colour Intensity in F_2_ Progeny in Gentian

**Figure 2:**
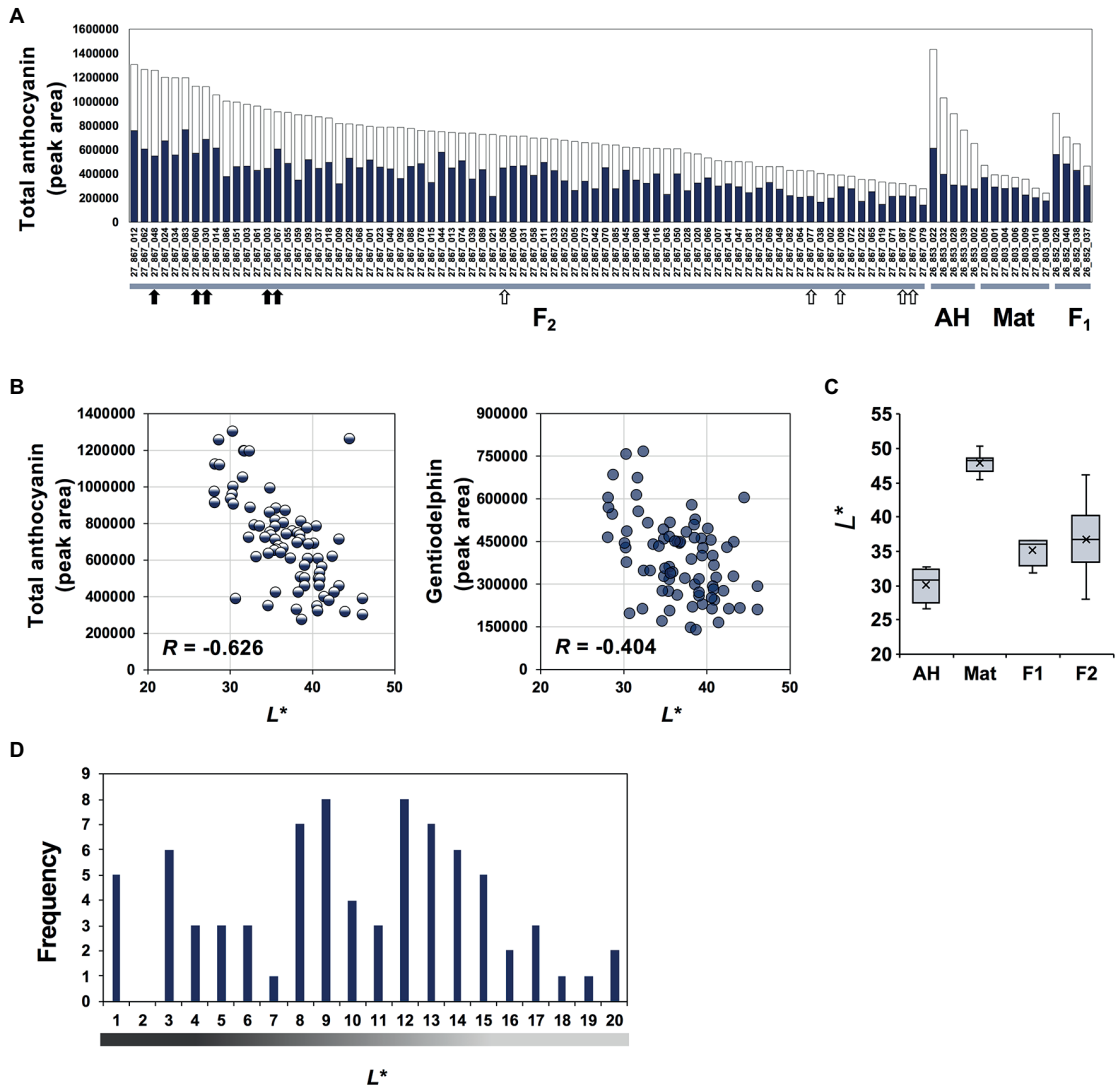
Anthocyanin content and L^*^ values for parents, F_1_ and F_2_ progeny. **(A)** Total anthocyanin content estimated from high-performance liquid chromatography peak area. Five AH, seven Mat, four F_1_ progenies 26-852 (AH × Mat), and 78 F_2_ progenies 27-867 are shown. Bar colour indicates peak areas of gentiodelphin (blue) and other anthocyanins (white). Black and white arrows indicate lines of the intense- and faint-blue flower groups subjected to RNA-seq analysis, respectively. **(B)** Plots of L^*^ values and total anthocyanin or gentiodelphin contents. **(C)** Box plot of L^*^ values of parental lines, F_1_ and F_2_ progeny. **(D)** Histogram of F_2_ plants. L^*^ values representing flower colour intensity are separated into 20 steps.

The appearance of flowers of AH, Mat, and their F_1_ line (26-852) can be seen in Figure 1A. AH has a more intense colour than Mat, and the F_1_ progeny shows intermediate colour intensity. Other F_1_ progeny showed a similar colour intensity to line (26-852). In contrast, F_2_ progeny showed various degrees of colour intensity. The HPLC profile of petal extracts (520 nm) from these samples showed one major peak for gentiodelphin and its intermediate metabolites. The typical result of AH is shown in Figure 1B. Based on these peak areas, the total anthocyanin and gentiodelphin contents in parental lines, AH (five plants), and Mat (eight plants), F_1_ line 26-852 (four plants), and F_2_ line 27-867 (78 plants) were estimated and are shown in decreasing order of total anthocyanin content (Figure 2A). The total anthocyanin content of AH flowers was, on average, 2.7-fold greater than that of Mat flowers. The total anthocyanin content of the F_1_ plants was intermediate between that of AH and Mat. F_2_ plants showed a range of anthocyanin contents that fell between the values for AH and Mat. The difference between the highest (27-867-012) and lowest (27-867-079) anthocyanin content in F_2_ plants was 4.7-fold. The gentiodelphin content (Figure 2A, blue bars) of F_2_ plants did not match perfectly with the total anthocyanin content, but showed a similar overall order.

Next, to objectively evaluate flower colour intensity, colorimetric measurement was performed on each flower petal. We used L^*^ values of the CIELAB colour space, which represents the darkness or lightness of the colour (black = 0 and white = 100), to quantify the visible flower colour intensity. L^*^ values corresponded well with visual observations of flower colour intensity. L^*^ values showed stronger correlations (*R* = −0.626) with total anthocyanin content than with gentiodelphin content (*R* = −0.404; Figure 2B), but both contributed to flower colour intensity. Box plots of L^*^ values of AH, Mat and their F_1_ and F_2_ seedlings are shown in Figure 2C. Compared with the L^*^ values of AH (average 30.08) and Mat (average 47.88), those of F_1_ were intermediate and closer to AH. F_2_ seedlings showed a wide range of intensities (range 28.07–46.08). The frequency distribution of L^*^ values among the 78 plants in F_2_ 27-867 is shown in Figure 2D. Among these F_2_ plants, five that showed lower L^*^ values (27-867-048, 27-867-060, 27-867-030, 27-867-003, and 27-867-067) and five that showed higher L^*^ values (27-867-056, 27-867-077, 27-867-008, 27-867-087, and 27-867-076) were selected for additional RNA-seq analysis. Flowers of these lines are shown in Supplementary Figure S1.

### Construction of Reference Sequence and Selection of DEGs Between Intense- and Faint-Blue Flowers Using RNA-Seq Analysis

As a result of RNA-sequencing for reference construction based on petals and/or leaves of AH, Mat, 26-852, IO and 26-850, a total of 743,968,608 high-quality 75–130 base length reads (83.4% of the raw data) were obtained. The reference was constructed using Trinity software and consisted of 148,061 contigs (annotated 50,948 contigs), showing N50 = 1,519 bp (Supplementary Table S2). These contigs were used as a reference for the construction of two types of expression profiles. All the raw sequence data for reference construction were registered to the DDBJ DRA database (accession no. DRA010021).

As a result of RNA-sequencing of S4 petals of each of the five F_2_ intense- and faint-blue flower lines, 6,595,446 high-quality, 75–130 base length reads (81.3% of the raw data) were obtained. All the raw sequence data of the F_2_ lines were registered to the DDBJ DRA database (accession no. DRA013625). Using these data and the reference described above, normalised expression profiles of the F_2_ lines were constructed using the RSEM and TMM softwares. Conversely, the normalised expression profiles of S1–S4 petals in AH and Mat were constructed using their 8,777,392 high-quality 75–130 base length reads (76.1% of the raw reads) by the method described above. All the raw sequence data were registered to the DDBJ DRA database (accession no. DRA014102). Information regarding RNA-sequencing, such as raw reads, processed reads, and accession, is summarised in Supplementary Table S1.

**Figure 3:**
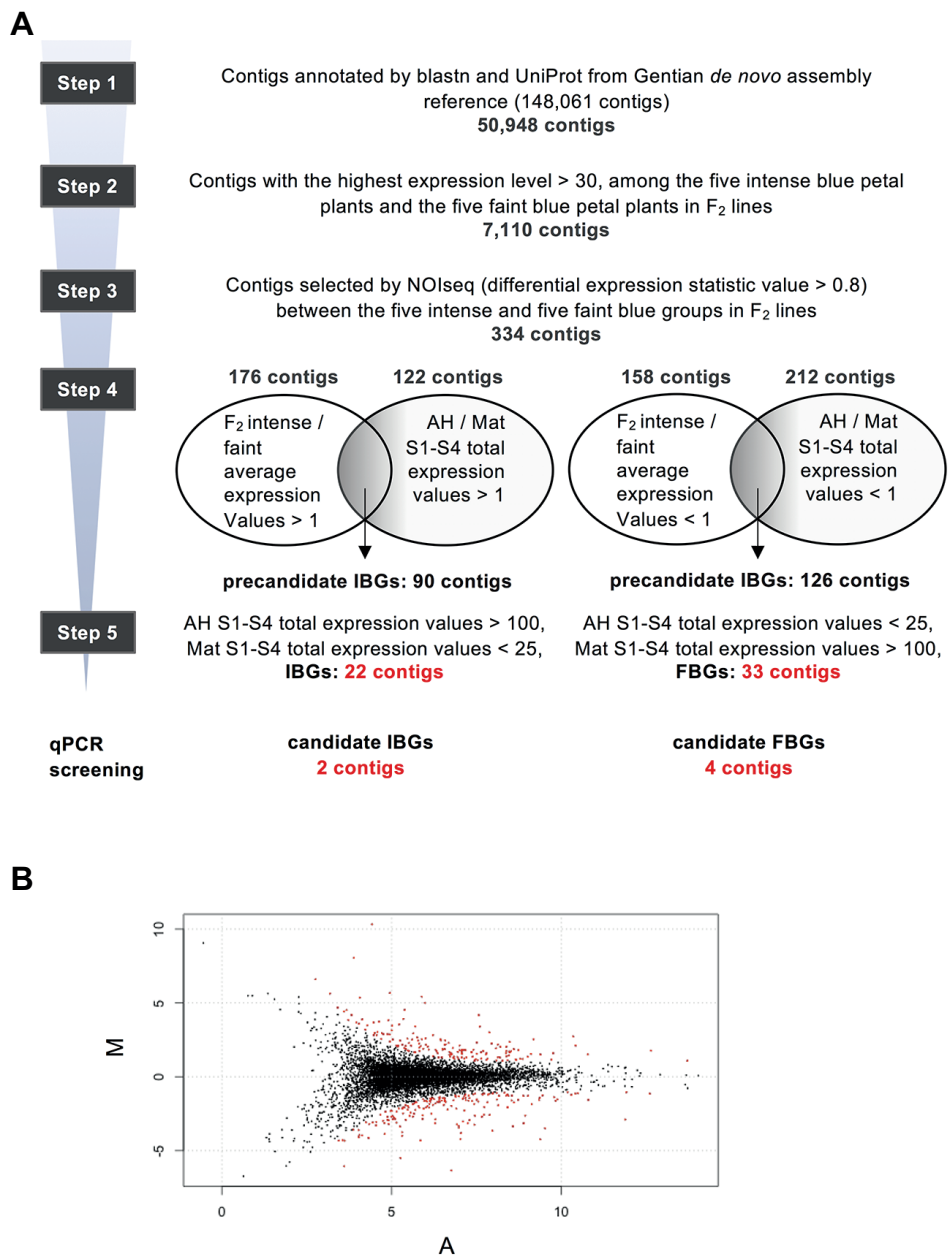
Schematic of screening procedure of differential expression genes (DEGs). **(A)** Screening procedures of the DEGs. **(B)** MA-plot of 7,110 contigs in step 2. Red plots are 334 contigs of differentially expression genes selected from the probability of differential expression estimated using NOISeq analysis (Tarazona et al., 2015), with parameter *q* > 0.8.

Then, we attempted to obtain DEGs between faint- and intense-blue flower lines. The screening procedure for DEGs based on the normalised expression profiles is summarised in Figure 3A. From 50,948 annotated contigs, 7,110 that showed high expression levels in F_2_ lines were selected. Using these, NOISeq analysis was performed and 334 contigs that satisfied the condition of values >0.8 were selected (Figure 3B). Although the NOISeq screening described above was performed using S4 petals of F_2_ progenies, in the next screening, S1–S4 total expression values of petals in AH and Mat were confirmed to consider the entire period of flower development.

There were 176 contigs that satisfied the condition of values >1 for the average expression levels of five intense-blue flower lines/average expression levels of the five faint-blue flower lines. Among these contigs, 90 were selected as precandidate intense-blue genes (IBGs), which satisfied the condition of values >1 for expressions levels for S1–S4-total for AH/S1–S4-total for Mat. There were 158 contigs that satisfied the condition of values <1 for the average expression levels of the five intense-blue flower lines/average expression levels of the five faint-blue flower lines. Among these, 126 contigs were selected as precandidate FBGs, which satisfied the condition of values <1 for S1–S4-total expression for AH/S1–S4-total expression for Mat.

From the precandidate 90 IBG contigs, 22 were selected as candidate IBGs using the following optional conditions: AH S1–S4 total expression value >100, Mat S1–S4 total expression value <25. In a similar fashion, from 86 precandidate contigs, 33 were selected as candidate faint-blue genes (FBGs) using the following conditions: AH S1–S4 total expression value <100, Mat S1–S4 total expression value >25. The top 10 selected contigs, for IBGs and FBGs, are shown in Table 1, and all contigs before and after the step 4 selection are shown in Supplementary Tables S4 and S5, respectively.

**Table 1:** List of the top 10 DEGs [intense-blue genes (IBGs) and faint-blue genes (FBGs)].

Type	Contig	Candidate function	NOISeq		Ratio of expression levels of intense blue group/faint blue group^y^	AH S1–S4 total expression values^z^	Mat S1–S4 total expression values^z^
			Statistics	Ranking			
IBG1	TRINITY_DN976_c0_g1_i1	Non-specific Lipid transfer protein	0.942	15	13.665	186.5	23.5
IBG2	TRINITY_DN43721_c0_g1_i1	Endglucanase	0.912	44	15.555	3398.0	2.7
IBG3	TRINITY_DN4921_c0_g2_i1	Desiccation protectant protein Lea14 homolog	0.886	91	6.116	330.6	0.0
IBG4	TRINITY_DN24389_c0_g1_i2	BAHD acyltransferase	0.880	109	3.848	3093.8	0.0
IBG5	TRINITY_DN5541_c0_g1_i1	Aquaporin PIP1-2	0.864	144	4.257	845.8	0.0
IBG6	TRINITY_DN23897_c0_g1_i1	GATA transcription factor 24	0.856	158	5.439	200.3	0.0
IBG7	TRINITY_DN19860_c0_g1_i1	Probable E3 ubiquitin-protein ligase HERC4	0.851	169	3.184	337.1	0.0
IBG8	TRINITY_DN20828_c0_g1_i2	Actin-depolymerizing factor 5-like	0.848	180	7.387	181.2	0.0
IBG9	TRINITY_DN38305_c1_g1_i3	Uncharacterised protein	0.834	224	16.546	325.5	0.0
IBG10	TRINITY_DN22028_c0_g1_i2	Probable protein phosphatase 2C	0.832	227	9.887	352.1	1.1
FBG1^x^	TRINITY_DN46412_c1_g1_i2	Mini zinc finger protein	0.994	1	0.001	1.4	1092.7
FBG2	TRINITY_DN59482_c0_g1_i1	Bidirectional sugar transporter SWEET3	0.975	3	0.004	0.4	237.4
FBG3	TRINITY_DN5486_c0_g1_i1	Expansin-like B1	0.959	4	0.023	12.7	262.7
FBG5	TRINITY_DN41577_c1_g1_i1	Alkane hydroxylase MAH1-like (CYP)	0.953	7	0.030	0.9	180.8
FBG6	TRINITY_DN23896_c1_g1_i1	Xyloglucan endotransglucosylase/hydrolase	0.951	9	0.055	17.3	326.5
FBG7	TRINITY_DN48951_c3_g2_i1	bHLH148-like	0.950	10	0.019	1.2	125.2
FBG8	TRINITY_DN46309_c0_g3_i1	Peroxidase P7-like	0.932	26	0.041	2.2	100.5
FBG9	TRINITY_DN40961_c0_g1_i2	Cystathionine gamma-synthase	0.925	34	0.000	4.8	412.9
FBG10	TRINITY_DN51157_c4_g1_i2	Solanesyl-diphosphate synthase 1	0.913	43	0.064	13.1	122.1
FBG11	TRINITY_DN27922_c0_g1_i1	Uncharacterised protein	0.905	56	0.024	4.7	103.9

x FBG4 (TRINITY_DN46412_c1_g1_i1) was removed because the sequence was similar to FBG1 (TRINITY_DN46412_c1_g1_i2) from target of analysis.

y Ratio = average of normalised expression values in petals of intense blue group (*n* = 5)/average of normalised expression values in petals of faint blue group (*n* = 5).

z S1–S4 total expression values means total normalised expression values in petals in flower development stages 1–4.

### Quantitative RT-PCR Analysis of the Candidate Genes

**Figure 4:**
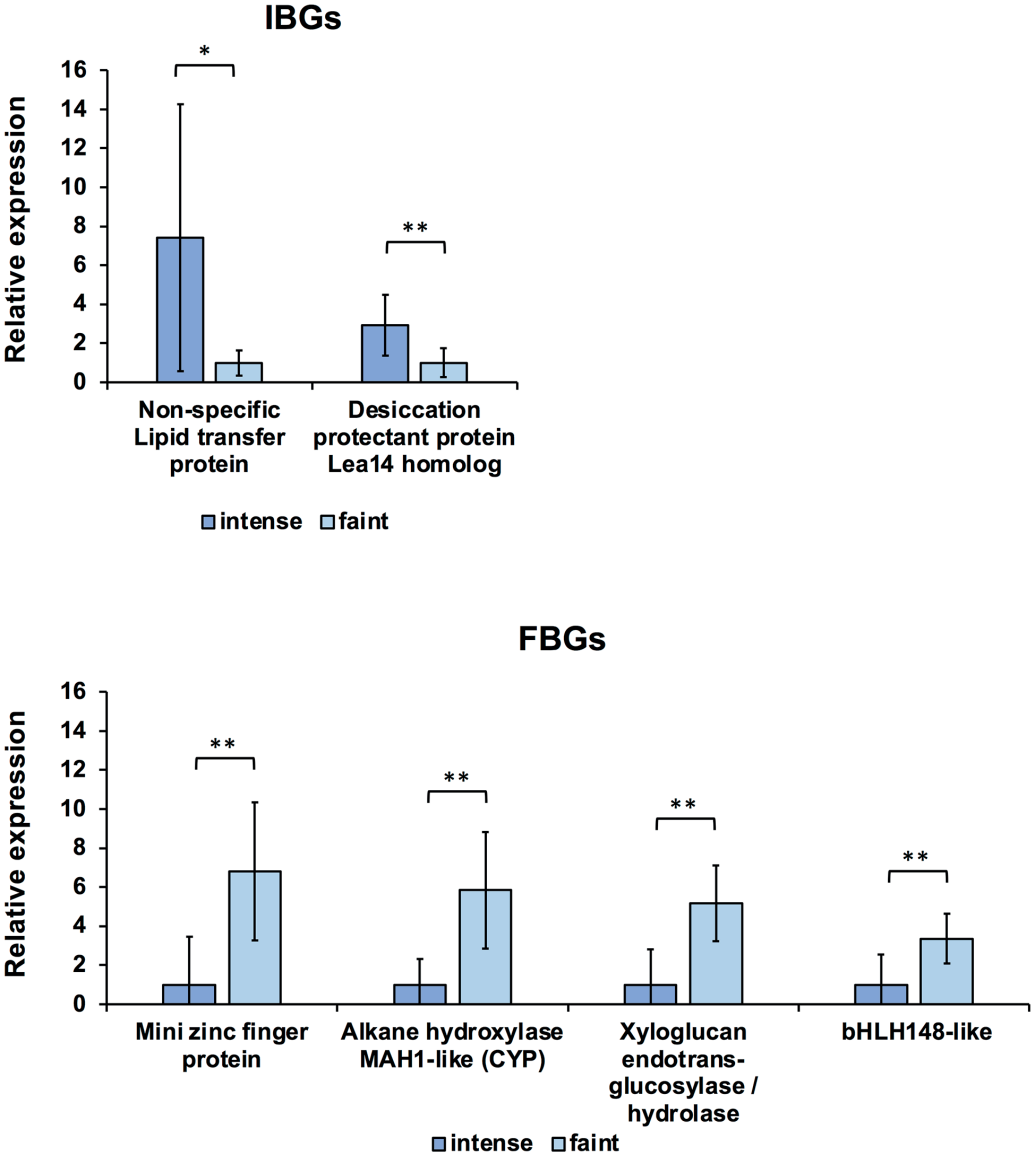
Quantitative RT-PCR (qRT-PCR) analysis of candidate intense-blue genes (IBGs) and faint-blue genes (FBGs) in petal stage 4 of intense- and faint-blue F_2_ individuals. Expression of each gene was normalised using *GtUBQ* as the internal control. Error bars indicate means ± SDs. * Shows statistical significance (Welch’s *t*-test at ^**^p < 0.01, ^*^p < 0.05).

To further narrow down the candidate genes, we performed qRT-PCR analysis for each of the top 10 IBG and FBG contigs screened by normalised expression data of RNA-seq. All contigs were expressed in the S4 petals of AH and Mat (Supplementary Figure S2). Therefore, S4 petals of the intense- and faint-blue flower groups in F_2_ progeny were used for qRT-PCR analysis. The designed primers are listed in Supplementary Table S3, and the screening workflow is summarised in Supplementary Table S6. The first screening was performed on each of the top 10 DEGs (Table 1) using F_2_ progeny of two intense-blue individuals and two faint-blue individuals. Among four patterns of the relative expression of the intense- (IB1, IB3) and faint-blue group plants (FB2, FB4) in F_2_ progenies, six and seven contigs that satisfied the conditions of >4.00 for IBG and <0.25 for FBG in at least three patterns were selected as candidates for IBG and FBG, respectively. In the second screening, two and five candidates for IBG and FBG were selected using Welch’s *t*-test (*p* < 0.05, *n* = 6) in IB and FB plants, respectively. Finally, in the third screening, two and four candidates for IBG and FBG were selected by Welch’s *t*-test (*p* < 0.05, *n* = 10) in 10 plants, including four more individuals added after the second screening. The expression levels of two candidate IBGs and four candidate FBGs and statistical differences between the intense- and faint-blue groups are shown in Figure 4. The two-dimensional scatter between gene expression levels and their corresponding L^*^ values showed different spatial distributions between the intense- and faint-blue groups (Supplementary Figure S3). We selected the first ranking gene from the NOISeq analysis, FBG1, which was a homolog of the MIF protein gene *GtMIF1*; this was subjected to further functional analysis.

### Cloning of *GtMIF1* and Homology Analysis

*GtMIF1-1* and *GtMIF1-2* were isolated from the F_2_ plant line that showed the lowest L^*^ value. cDNAs of *GtMIF1-1* and *GtMIF1-2* have been registered in a public database under accession numbers, LC699354 and LC699355, respectively. Alignment with other plant MIF proteins shows that the deduced amino acid sequences of GtMIF1-1 and GtMIF1-2 contain a conserved zinc-finger (ZF) domain (CX_3_HX_11_CX_12–26_CX_2_CXCHX3H) in which five cysteine and histidine residues (Hu and Ma, 2006) are completely conserved (Supplementary Figure S4). GtMIF1-1 and GtMIF1-2 are considered as allelic because they have high similarity (98.3%) and were cloned from F_2_ progeny. The 3′-terminus and 3′-UTR of GtMIF1-1 and GtMIF1-2 was different (Supplementary Figure S5). Phylogenetic analysis, based on amino acid sequences, showed that both GtMIF1-1 and GtMIF1-2 are close to rice OsMIF3, OsMIF4, and maize ZmMIF4 (Supplementary Figure S6). In terms of dicotyledonous plants, GtMIF1-1 and GtMIF1-2 are close to cotton (GhiMIF2) and tomato (SlIMA).

### Production and Phenotype Analysis of Tobacco Transformants Overexpressing *GtMIF1*

**Figure 5:**
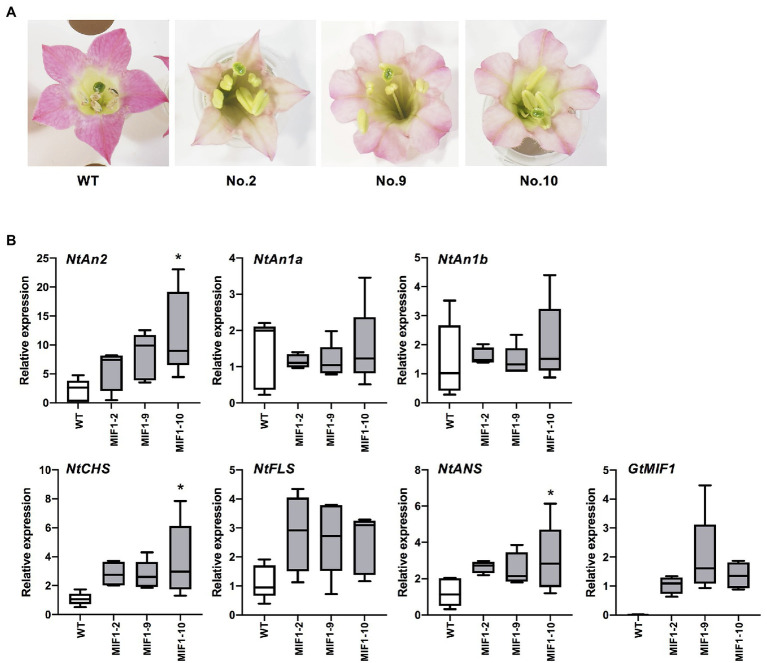
Transgenic tobacco plants overexpressing *GtMIF1*. **(A)** Typical flowers of WT and three T_2_ lines. **(B)** qRT-PCR analysis of flavonoid biosynthesis-related genes in petals. Relative expression levels are shown using tobacco *UBQ* gene as an internal reference. * Indicates a significant difference between the expression levels of *GtMIF1*-ox lines and those of WT (Welch’s *t*-test at ^*^p < 0.05).

Japanese gentian is difficult to stably transform, except particular cultivars; therefore, we used tobacco to gain insights into the function of *GtMIF1*. Transgenic tobacco plants (*Nicotiana tabacum* L.) expressing *GtMIF1* were produced *via Agrobacterium*-mediated transformation using the binary vector pSKAN35SGtMIF1. Eleven kanamycin-resistant lines were obtained and grown in a closed greenhouse. Some plants showed abnormal growth; 10 lines set flowers (Supplementary Figure S7A). *GtMIF1* expression levels in the leaves were determined by qRT-PCR analysis. Primary transformants with strong *GtMIF1* expressing lines (nos. 3–6) showed reduced growth and morphological aberrations, such as wavy leaves, and these plant lines were infertile. Flower colours tended to be lighter, except for line no. 7. Finally, seven lines set seeds by self-pollination and from these we selected three (nos. 2, 9 and 10). T_1_ seeds of these three lines were sown on kanamycin-containing medium, and kanamycin-resistant transgenic plants were grown in a closed greenhouse for further detailed analysis. The plants showed reduced height and slightly wavy leaves compared with untransformed WT plants (Supplementary Figure S8). The flower colours of these three lines were paler than those of WT plants (Figure 5A; Supplementary Figure S7B). To analyse the effect of *GtMIF1* overexpression on tobacco flowers, we performed qRT-PCR analysis of several endogenous genes involved in flavonoid biosynthesis. *GtMIF1* expression was confirmed in T_1_ flowers, and the expression levels were similar among the three lines. Expression levels of the structural genes (*NtCHS*, *NtANS*, and *NtFLS*) and transcription factor genes (*NtAn2*, *NtAn1a*, and *NtAn1b*) responsible for flavonoid biosynthesis in tobacco flowers were not significantly different from WT, except for *NtCHS*, *NtANS* and *NtAn2* of line no. 10 (Figure 5B).

### Analysis of Inheritance of *GtMIF1* by DNA Marker and L^*^ Value

**Figure 6:**
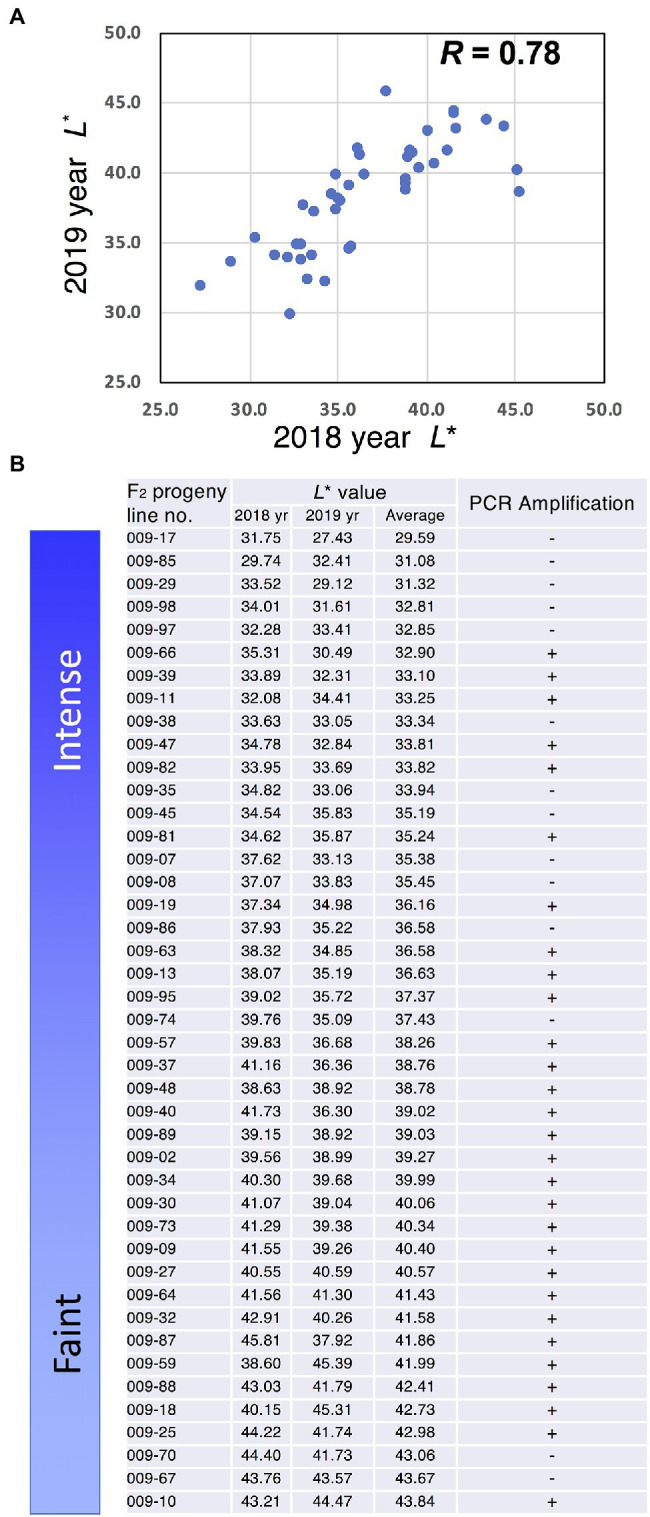
Relationship between *GtMIF1* and L^*^ value. **(A)** L^*^ value plots for 2 years. **(B)** PCR analysis of the *GtMIF1* allele associated with faint-blue colour in F_2_ progeny.

Japanese gentians are perennial plants; therefore, we analysed the stability of flower colour intensity for 2 years. Measurement of L^*^ values in F_2_ progeny indicated a strong correlation (*R* = 0.78) between years (Figure 6A). To analyse the relationship between flower colour intensity, represented by L^*^, and *GtMIF1*, we analysed these F_2_ progeny using genomic PCR targeted for *GtMIF1*. We designed PCR primers specifically amplifying a *GtMIF* allele in the faint-blue flower line. This DNA marker was amplified in individuals with both homozygous and heterozygous *GtMIF1* faint alleles but was not amplified in individuals with homozygous *GtMIF1* intense allele. PCR amplification was observed in 68 of the 83 individuals analysed (Figure 6B; Supplementary Figure S9), matching the expected frequency of a 3:1 segregation (chi-squared test, *p* < 0.05), suggesting that *GtMIF1* was inherited as a single locus. The PCR amplifications were predominantly observed in individuals with faint colour intensity, except for two lines (009-70 and 009-67; Figure 6B).

## Discussion

Ornamental flowers show various flower colours such as red, blue, and yellow, determined by plant pigments such as flavonoids, carotenoids, and betalains (Tanaka et al., 2008). The main pigments differ greatly among plant species and even among cultivars. Even with the same type of pigment, flower colour shows variation. Both the quality and quantity of accumulated pigments are important for the determination of flower colour, and, in some cases, they are responsible for pattern formation.

Among the flower pigments, flavonoids are the most well known. The chemical structures of approximately 8,000 kinds of flavonoids, including coloured anthocyanins, have been characterised to date (Iwashina, 2015). Although the accumulated pigments and their biosynthetic pathways are well characterised, there are few reports on regulation of the quantity of pigments in flowers. Differences in the expression levels of structural genes are likely responsible for flower colour intensity. The underlying mechanism may be differences in the promoter of each gene or the expression levels of transcription factors responsible for the relevant genes. The MYB-bHLH-WDR complex is involved in flavonoid biosynthesis (Xu et al., 2015), and MYBs are the most characterised transcription factor genes related to anthocyanin biosynthesis (Yan et al., 2021). In *P. ostia*, two transcription factors, PoMYB2 and PoSPL1, negatively regulate anthocyanin accumulation (Gao et al., 2015). Genes responsible for anthocyanin transport also reportedly affect flower colour. In carnation, *DcGSTF2*, encoding glutathione *S*-transferase-like gene, is responsible for flower colour intensity (Sasaki et al., 2012). In Japanese cultivated gentian, genes involved in flavonoid biosynthesis and transcriptional regulation have been characterised (Nakatsuka et al., 2014), but no genes related to regulation of flower colour intensity have been identified.

Here, we hypothesised that previously characterised structural and/or transcription factor genes are the key genes responsible for the variation in Japanese gentian flower colour intensity between intense-blue (Ashiro-no-Hatsuaki) and faint-blue (Matsuo) lines. However, RNA-seq analyses revealed that the expression levels of structural and transcription factor genes involved in flavonoid biosynthesis were unclear for the different flower colour intensities (Supplementary Figure S10), indicating the existence of another relevant gene(s). To identify the genes responsible, we produced progenies and analysed the inheritance of flower colour intensity. The F_1_ plants showed intermediate flower colour intensity and F_2_ plants obtained from self-pollination of one F_1_ plant (26-852-20) showed varying colour intensity, as indicated by L^*^ values (Figure 2D). This suggests that flower colour intensity in gentian is a quantitative trait locus (QTL). The QTL of flower colour intensity based on anthocyanin amount has been investigated in other plant species, such as rose (Henz et al., 2015) and *Mimulus* (Yuan et al., 2013). It is also known that the allele situation of the flavonoid biosynthetic and regulatory genes affects the flower colour intensity. The heterozygote of functional/non-functional alleles generally shows fainter flower colour than that of functional alleles, although several examples of single-gene overdominance were reported (Liberatore et al., 2013). Besides anthocyanin biosynthesis, branching biosynthetic pathways to other flavonoid classes, such as flavones and flavonols, can also have quantitative effects on the colour intensity (Luo et al., 2015; LaFountain et al., 2017). However, except for pigment biosynthesis-related genes, genes that are quantitatively related to flower colour intensity have not been clarified. We focused on an FBG1 (MIF protein homolog) because it was the highest-ranking contig from the NOISeq analysis. Based on individual plants, FBG1 was selected as a possible candidate gene by two-dimensional scatter plot (Supplementary Figure S3).

We cloned two genes, *GtMIF1-1* and *GtMIF1-2*, encoding putative small zinc-finger proteins that show a highly conserved MINI ZF domain in plants (Supplementary Figure S4). In *Arabidopsis*, *AtMIF1* and *AtMIF3* are expressed in vegetative organs, and *AtMIF2* is expressed in the floral meristem (Hu and Ma, 2006; Winter et al., 2007). *AtMIF1* is related to vascular development, and its overexpression using the CaMV35S promoter revealed pleiotropic defects of plant development, such as dwarfism, dark-green leaves, and altered flower morphology (Hu and Ma, 2006). AtMIF2 and its tomato homolog, S1IMA, are reported to regulate floral meristem termination, and their overexpression also induced severe reductions in plant and flower size (Bollier et al., 2018). Our results also revealed that overexpression of *GtMIF1* in tobacco caused morphological aberrations, including reduced plant height and wavy leaves. Plant lines with strong expression showed extreme phenotypes and were infertile (Supplementary Figure S7A). Although GtMIF1-1 and GtMIF1-2 were not classified in the same clade as AtMIFs and S1IMA in the phylogenetic tree, our results support the hypothesis that GtMIF1 has a similar function to AtMIFs and S1IMA.

In previous studies of Arabidopsis and tomato, the effects of *MIF* genes on flower colour were not examined. However, our results clearly showed a reduction in flower colour intensity in *GtMIF1*-overexpressing tobacco flowers (Figure 5A), suggesting that *GtMIF1* also affects flower pigmentation. Because a constitutive 35S promoter was used for the ectopic expression of *GtMIF1* in tobacco plants, the phenotype of dwarf or wavy leaves may have been observed. In future studies, transgenic studies using own or petal-specific promoters are necessary to reveal the exact function of GtMIF1 in gentian petals. Moreover, promoter analysis between two *GtMIF1* alleles would provide firm evidence of the contribution of GtMIF1 in flower colour intensity regulation. In the future, we would like to clarify the molecular mechanism of flower colour intensity regulation by *GtMIF1*.

In tobacco *GtMIF1*-overexpressing lines, the expression levels of flavonoid biosynthetic genes and transcription factor genes did not differ overall and fell within the expected variation (Figure 5B). Although a significant difference in the expression of some genes was observed in line MIF1-10, the expression levels of foreign *GtMIF1* were comparable among the three transgenic lines, indicating that the activated expressions of *NtAn2*, *NtCHS* and *NtANS* were not responsible for the variation in flower colour. Moreover, these three genes usually increase anthocyanin accumulation; therefore, it is unlikely that reduced flower colour would result from activation of these genes. In gentian, there were also no notable differences in the expression levels of known flavonoid-related genes between the two parental lines (Supplementary Figure S10). These findings suggest that direct regulation of flavonoid biosynthetic genes is not responsible for differences in flower colour intensity. It is likely that hormonal changes are responsible for flower colour reduction because dwarf and morphological aberrations were observed (Supplementary Figures S7C, S8). In *Gerbera hybrida*, a MIF homolog protein inhibited petal elongation by activating the GASA protein family gene, GEG (Han et al., 2017). In that study, decreases and increases in flower pigmentation were observed in *GhMIF*-overexpressed and *GhMIF*-VIGS lines, respectively, although the analysis was not focused on petal colour. Arabidopsis *AtMIF1* gene is known to affect the development of various plants *via* hormone network pathways; therefore, it is likely that hormone-mediated signalling pathways contribute to flower colour intensity variation in gentian. In *Arabidopsis*, it is difficult to study the involvement of *AtMIF* genes in flower colour regulation, because *Arabidopsis* have colourless petals.

Next, to analyse whether *GtMIF1* was involved in the regulation of flower colour intensity in gentian, we developed a PCR-based DNA marker and analysed the F_2_ population for 2 years. The strong correlation between years indicated that flower colour intensity was comparatively stable and plant age did not affect the phenotype. Analysis of the DNA marker confirmed that *MIF1* was inherited as a single locus and correlated well with faint colour intensity (Figure 6). We previously developed DNA markers to distinguish blue from pink or white flowers (Kakizaki et al., 2009; Nakatsuka et al., 2011). In those cases, the responsible genes were functional specific genes such as *F3′5′H*, *MYB3* and *ANS*; therefore, the concordance rate was almost 100% between genotype and phenotype. However, in this study, the PCR marker of *MIF1* did not perfectly match the faint flower phenotype. The loss of *MIF1* linkage observed in F_2_ (e.g., lines 009-70 and 009-67 in Figure 6B) suggests that other genes also play a role in the determination of flower colour intensities. One possible reason of the segregation inconsistency of *MIF1* allele is a recombination event; in this case, neighbouring *GtMIF1* may be alternative candidates for the trait. Several genes are also predicted in genome contigs containing *GtMIF1* (Supplementary Figure S11). GtMYBP9 may be a candidate because it is near to AmMIXTA and MIXTA-like proteins that regulate epidermal cell shape and pigmentation in *Antirrhinum majus* (Baumann et al., 2007). Further analysis is necessary to completely understand the regulation mechanism of flower colour intensity in Japanese gentian.

Among the selected FBGs, FBG5, FBG6, and FBG7 were annotated as putative alkane hydroxylase MAH1-l like (CYP), xyloglucan endotransglucosylase/hydrolase, and bHLH148-like, respectively. As far as we know, there are no reports on the involvement of these genes in anthocyanin biosynthesis. Two putative peroxidases, FBG8 and FBG15, were also included in the list (Supplementary Table S5). The mechanism of anthocyanin degradation is poorly understood, but the involvement of peroxidase is suspected in several plant species. In *Brunfelsia calycina* flowers, a vacuolar class III peroxidase, encoded by *BcPrx01*, was identified as a candidate gene for changes in colour from purple to white. *BcPrx01* expression and activity in petals were associated with the degradation of anthocyanins (Zipor et al., 2015). Furthermore, grapevine *VviPrx31*, encoding a putative peroxidase, is reportedly a candidate gene of anthocyanin degradation in ripening berries, although its activity has not been confirmed (Movahed et al., 2016). In paprika, enzymatic degradation of anthocyanins is likely responsible for fading of colouration in fruit (Yamada et al., 2019). Phylogenetic analysis indicated that FGB8 and FBG15 were close to *BcPrx01* (Supplementary Figure S12); therefore, it is likely that these genes are involved in anthocyanin degradation in gentian flowers. We could not find any relevant references about other selected DEGs; therefore, further analysis is necessary to assess their contribution to flower colour intensity in gentian.

In conclusion, we identified candidate genes responsible for flower colour intensity in Japanese gentian. Functional analysis of *GtMIF1* confirmed that this gene has the ability to reduce flower colour in heterologous tobacco plants. Progeny analysis using a *GtMIF1* DNA marker revealed that this gene is involved in flower colour intensity in F_2_ individuals. Thus, it is likely that *GtMIF1* is one of the key genes that determine flower colour intensity in gentian. Environmental conditions such as light and temperature are known to affect anthocyanin accumulation in plants (Azuma et al., 2012). High temperature in the field or dim light during transport of cut flowers also influences flower colour intensity in gentian. For breeding of stable colour cultivars, further analyses of these environmental factors, as well as genetic features, are necessary.

## Data Availability Statement

The datasets presented in this study can be found in online repositories. The names of the repository/repositories and accession number(s) can be found at: DDBJ—DRA013625, DRA010021, DRA014102, LC699354, and LC699355.

## Author Contributions

KT and MN conceived the original research plan and wrote the manuscript. AW, FG, and NS performed sampling, RNA and DNA extractions, and measurement by spectrophotometer. KT performed RNA-seq and bioinformatic analyses. AW, KN, and ST performed qRT-PCR analyses. KN, AW, and MN performed *MIF1* cloning and DNA-marker analyses. TH performed crossing and cultivated gentian population. KT and NS performed HPLC analysis. All authors contributed to the article and approved the submitted version.

## Funding

This work was financially supported by commissioned projects, ‘Breeding of floricultural plants adapted for high practical needs and development of low cost cultivation techniques’ of the Ministry of Agriculture, Forestry and Fisheries, Japan.

## Conflict of Interest

The authors declare that the research was conducted in the absence of any commercial or financial relationships that could be construed as a potential conflict of interest.

## Publisher’s Note

All claims expressed in this article are solely those of the authors and do not necessarily represent those of their affiliated organizations, or those of the publisher, the editors and the reviewers. Any product that may be evaluated in this article, or claim that may be made by its manufacturer, is not guaranteed or endorsed by the publisher.
