# Supplementary material for: Identification of Candidate Genes Responsible for Flower Colour Intensity in Gentiana triflora
Source: Front Plant Sci. 2022 Jun 22;13:906879. doi: 10.3389/fpls.2022.906879 (PMC9257217; doi:10.3389/fpls.2022.906879)
Supplement: Supplementary file 1 [file Data_Sheet_1.PDF]

Supplementary Material

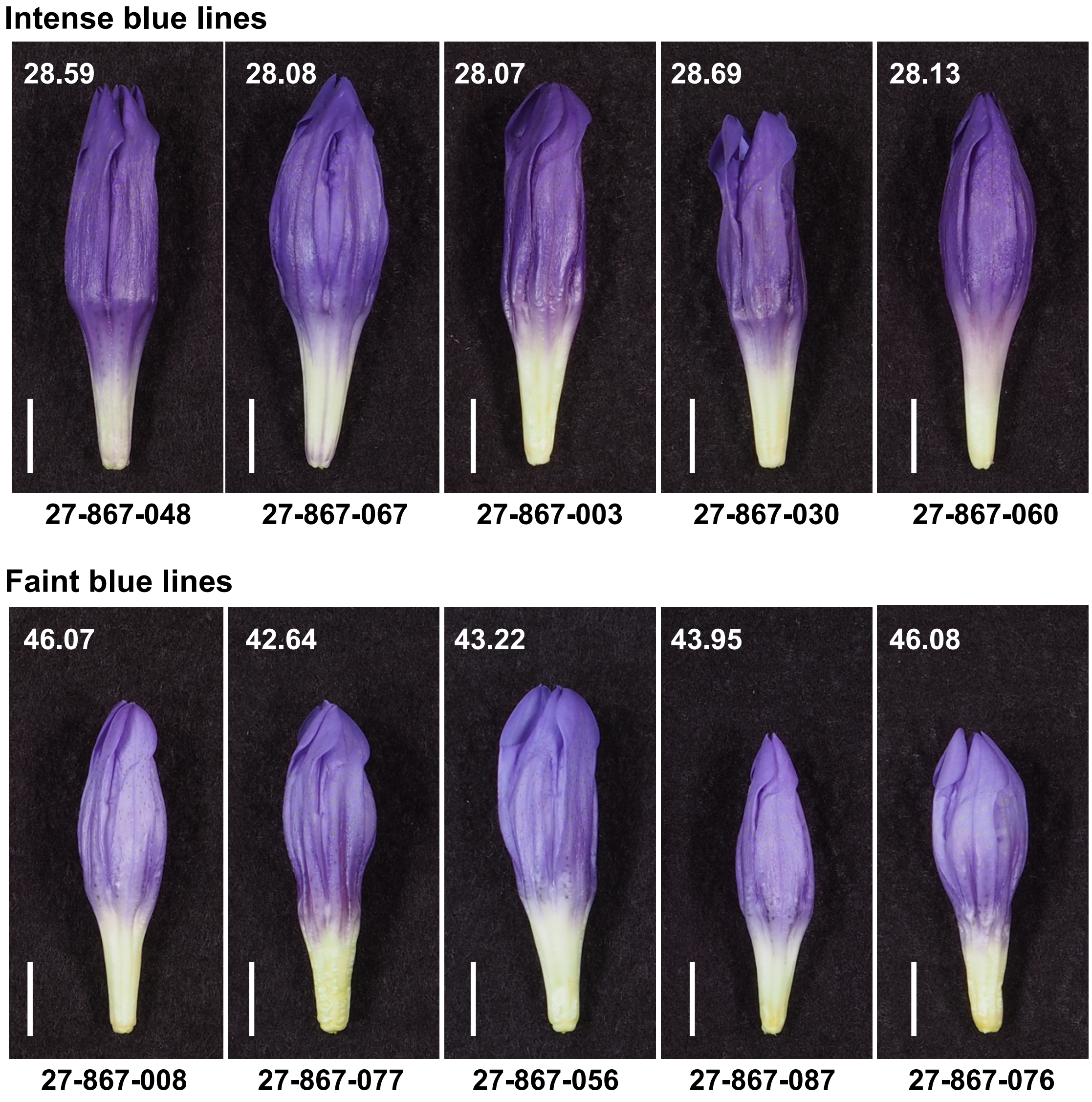

Figure S1. Flower photographs of AH × Mat F_2 plants (27-867) applied for RNA-sequencing analysis.

The values in the upper left of the each photo indicate *L** values measured on the adaxial surface of the petals. Scale bar = 1 cm.

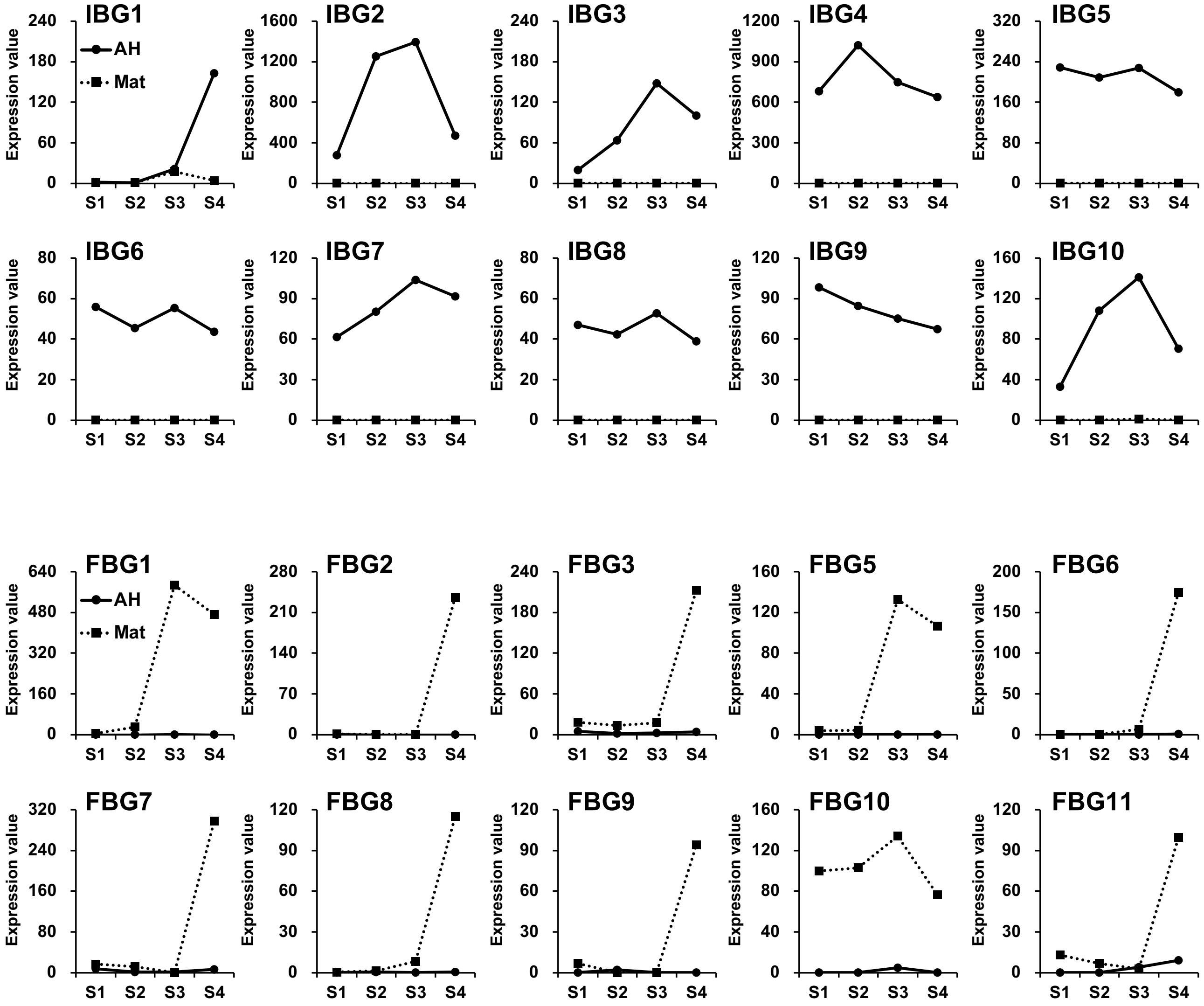

Figure S2. Expression patterns of the top ten contigs of intense-blue genes (IBGs) and faint-blue genes (FBGs) selected from normalised expression data from RNA-sequencing.

Data at four flower developmental stages (S1-S4) are shown. Expression values are calculated by TMM normalization from FPKM values.

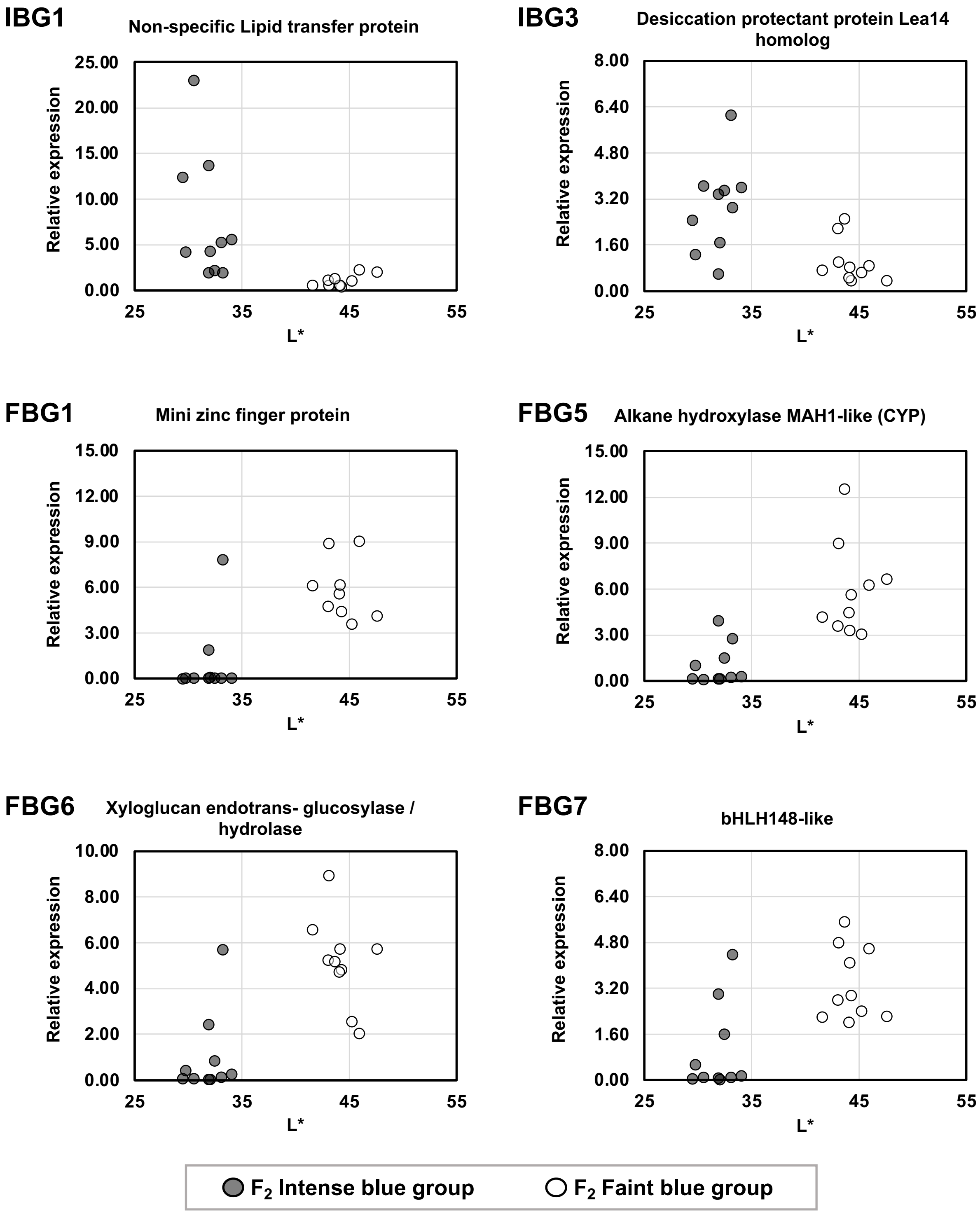

Figure S3. Visualisation of the two-dimensional scatter plot of *L values of the adaxial petal surface and expression levels of selected genes in flowers of F_2 individuals of intense- and faint-blue flower colour groups.**

```
AtMIF1          ----------------------------------MMKKRQMVIKQRS-RNSNTSSSWTTT 25
AtMIF3          -----------------------------------MKKRQVVIKQR-------KSSYTMT 18
GmMIF3          -----------------------------------MKKRQVVVKSV---------ANTSS 16
MtrMIF          -----------------------------------MKKRQVVAK-------------TSS 12
VvMIF1          -----------------------------------MKKRQIVVRRD--RSER--SSDTSS 21
RcMIF3          -----------------------------------MKKQQVVVRRD--GSSRRSSSSTSS 23
GhiMIF1         -----------------------------------MKKCQVVRKSG-------RRSCTSS 18
PtMIF2          MPASNYRSVFQLESSSLVYQGFERGEGSVELGTSKMRKRQVAVRRT---EEPSRSS-T-T 55
RcMIF2          -----------------------------------MRKRQVVVRR----EEPS------- 14
VvMIF2          MPASDSGSLEELENCSCFLG-GCGEEGRVDLGSCRMRKRQVVLRR----DEPSRSS-ANS 54
GmMIF2          -----------------------------------MRKRQVVVRR----EEPQRS----- 16
AtMIF2          -----------------------------------MRKRQVVLRRAS-PEEPSRSSSTAS 24
SiMIF2          -----------------------------------MKKRQVVLKR----DDQFSSNFANS 21
GhiMIF2         ----------------------------MIKGLAIMRRRKVVVRK----EEPPRKTTTNS 28
SlIMA           -----------------------------------MKK---VLRR----NDYSR-NSTNS 17
OsMIF2          ------------------------------------------------------------
ZmMIF1          -----------------------------------MGPQQGRRSNG---GAAARSKQEEE 22
OsMIF1          ------------------------------------------------------------
GtMIF1-1        -----------------------------------MKKLH---------LVAKRESSSSS 16
GtMIF1-2        -----------------------------------MKKLH---------LVAKRESSSSS 16
OsMIF4          ------------------------------------------------------------
ZmMIF4          -----------------------------------MMKRMVIVRRCHPPPPPPV--LLFG 23
OsMIF3          ------------------------------------------------------------
GhMIF           -----------------------------------MTKRRVVLKREEPPPPPPPSNPSMA 25


AtMIF1          SSSSSSSEISNVRYVECQKNHAANIGGYAVDGCREFMAAGV----------EGTVDALRC 75
AtMIF3          SSSS------NVRYVECQKNHAANIGGYAVDGCREFMASGG----------D---DALTC 59
GmMIF3          SVMR------NIRYGECQKNHAANIGGYAVDGCREFMASR-----------EGAGGALTC 59
MtrMIF          SITR------NIRYGECQKNHAANIGGYAVDGCREFMASTG----------EGTSGALTC 56
VvMIF1          STYR------SVRYRECQKNHAANMGGHAVDGCREFMASGV----------EGTSAAFTC 65
RcMIF3          SMVR------TVRYGECQKNHAANIGGYAVDGCREFMASGQ----------EGTAEALTC 67
GhiMIF1         STAIT-----NVRYGECQKNHAANIGGYAVDGCREFMASDV----------EGTTGALTC 63
PtMIF2          SFTIR-----NVKYGECLKNHAASVGGYAVDGCREFMASGE----------EGTADALTC 100
RcMIF2          SNTIR-----NVRYVECQKNHAAGVGGYAVDGCREFMASGE----------EGTA-ALTC 58
VvMIF2          SFTVR-----SVRYGECQKNHAAGVGGYAVDGCREFMASGE----------EGTSSALTC 99
GmMIF2          --GVR-----AVKYGECQKNHAANVGGYAVDGCREFMASGSGSGGGSGGG-EGTSAALTC 68
AtMIF2          SLTVR-----TVRYGECQKNHAAAVGGYAVDGCREFMASRGE---------EGTVAALTC 70
SiMIF2          SFAVR-----RVRYGECQKNHAANVGGYAVDGCREFMPCGE----------EGSNGALTC 66
GhiMIF2         SLTIT-----TVRYGECQRNHAASSGGYVVDGCREFMASGE----------EGTSGALAC 73
SlIMA           SFTMR-----RVRYVECQRNHAASVGGYVIDGCREYMPEG------------TTSGTLNC 60
OsMIF2          -------------YRECQRNHAASIGGHAVDGCREFMASGA----------DGTAAALLC 37
ZmMIF1          GGAKV------VRYRECQRNHAASLGGHAVDGCREFMAAGA----------DGTAAALAC 66
OsMIF1          -------------YRECQRNHAASIGGHAVDGCREFMASGA----------EGTAAALLC 37
GtMIF1-1        SSIIT------VRYMECQKNHAASIGGHVVDGCREFIASND----------ERENSAVMC 60
GtMIF1-2        SSIIT------VRYMECQKNHAASIGGHVVDGCREFIASND----------ERENSAVMC 60
OsMIF4          -------------YGECRRNHAARMGGHAVDGCREFLAEGEEG----------TGGALRC 37
ZmMIF4          GCPSAG----GVRYGECRRNHAASMGGHAVDGCREFLAEGEEG----------TAAVLHC 69
OsMIF3          -------------YGECRRNHAASTGGHAVDGCREFIAAEDGGGGNSTSAVGVAAAALKC 47
GhMIF           SANSSNTIRVHVRYGECQRNHAANVGGYAVDGCREFMAANAGTGDEEEE----TDGTLTC 81
                             * ** :****  **:.:*****::.                  .. *

AtMIF1          AACGCHRNFHRKEVDTEVVCEYSPPNA------------------------------- 102
AtMIF3          AACGCHRNFHRREVDTEVVCEYSPPNANN----------------------------- 88
GmMIF3          AACGCHRNFHRREVNTEVVCEYSPPNSGR----------------------------- 88
MtrMIF          AACGCHRNFHRREVQTEVVCEYSPPNYSR----------------------------- 85
VvMIF1          AACGCHRNFHRKEVDSEVVCDSS----------------------------------- 88
RcMIF3          AACGCHRNFHRREVETEVVCEYTPPNNSYQQ--------------------------- 98
GhiMIF1         AACGCHRNFHRREVETEVLCEYT----------------------------------- 86
PtMIF2          AACGCHRNFHRREVETEVICDCSSPSSNGN---------------------------- 130
RcMIF2          AACGCHRNFHRREVET--VCECSSPTSTEA---------------------------- 86
VvMIF2          AACGCHRNFHLREVETELVCECSSPTSNGK---------------------------- 129
GmMIF2          AACGCHRNFHKRQ-EAEVVSECSSPTSNGT---------------------------- 97
AtMIF2          AACGCHRSFHRREIETEVVCDCNSPPSTGN---------------------------- 100
SiMIF2          AACGCHRNFHRREEEAEVVCDGSSPTT------------------------------- 93
GhiMIF2         AACGCHRNFHRREVETEVVSECSSPNNSSSRA-------------------------- 105
SlIMA           AACGCHRNFHRREVETDVASECTSASSTTK---------------------------- 90
OsMIF2          AACGCHQSFHRRE--------------------------------------------- 50
ZmMIF1          AACGCHRSFHRREVEQPAADCDCDCSSTTSGA-------------------------- 98
OsMIF1          AACGCHRSFHRRE--------------------------------------------- 50
GtMIF1-1        AACGCHRSFHRR-VEEARAG-------------------------------------- 79
GtMIF1-2        AACGCHRSFHRR-VEEARAVSD------------------------------------ 81
OsMIF4          AACGCHRSFHRRV--------------------------------------------- 50
ZmMIF4          AACGCHRSFHRRMVQRSCCFCCDSADVAVAAAAAAAAAAERWDDDCSPESSASSSTPR 127
OsMIF3          AACGCHRSFHRRV--------------------------------------------- 60
GhMIF           AACGCHRNFHRRVVETE------------------------------------PSSP- 102
                ******:.** :
```

Figure S4. Alignment of MINI ZINC-FINGER proteins.

Frame means zinc finger (ZF) domain using CLUSTAL 2.1. IMA, INHIBITOR OF MERISTEM ACTIVITY. Sl, *Solanum lycopercicum*; At, *Arabidopsis thaliana*; Pt, *Populus trichocarpa*; Gh, *Gerbera hybrida*; Gm, *Glycine max*; Vv, *Vitis vinifera*; Si, *Sesamum indicum*; Ghi, *Gossypium hirsutum*; Mtr, *Medicago truncatula*; Os, *Oryza sativa*; Zm, *Zea mays*; Rc, *Rosa chinensis*.

GtMIF1-1
GtMIF1-2

```
  1' CGGATTAATT AGTCGAAGCA ATTGTTTAAT TAAATAGATT ATTACTATAT ATAATTAAGT AAACTTTTTA
     ********** ********** ********** ********** ********** ********** **********
  1" CGGATTAATT AGTCGAAGCA ATTGTTTAAT TAAATAGATT ATTACTATAT ATAATTAAGT AAACTTTTTA

 71' AAAAGAGAAA AACCATCCAC TATATATACA CACATAAGTA TCACTTCAAA CTAGGGTTAG GGTTTTCTAT
     ********** ********** ********** ********** ********** ********** **********
 71" AAAAGAGAAA AACCATCCAC TATATATACA CACATAAGTA TCACTTCAAA CTAGGGTTAG GGTTTTCTAT

141' CTTCTTAATA ATTAAAGCCT AATTAATCTC CCTATTATTA TTCAGCTGCC TTAAATAAAT ACAGAGAGAG
     ********** ********** ********** ********** ********** ********** **********
141" CTTCTTAATA ATTAAAGCCT AATTAATCTC CCTATTATTA TTCAGCTGCC TTAAATAAAT ACAGAGAGAG

211' AGAGAGAGAC TATAAATTAA AGCTATATAT AAATAATAAT GAAGAAACTC CATCTAGTGG CCAAGAGAGA
     ********** ********** ********** ********** ********** ********** **********
211" AGAGAGAGAC TATAAATTAA AGCTATATAT AAATAATAAT GAAGAAACTC CATCTAGTGG CCAAGAGAGA

281' GTCTTCTTCT TCTTCTTCTT CAATAATAAC AGTGAGATAC ATGGAGTGCC AGAAAAACCA TGCTGCAAGC
     ********** ********** ********** ********** ********** ********** **********
281" GTCTTCTTCT TCTTCTTCTT CAATAATAAC AGTGAGATAC ATGGAGTGCC AGAAAAACCA TGCTGCAAGC

351' ATAGGGGGTC ATGTTGTTGA TGGTTGCAGA GAGTTCATAG CTTCTAATGA TGAGAGAGAA AACTCTGCAG
     ********** ********** ********** ********** ********** ********** **********
351" ATAGGGGGTC ATGTTGTTGA TGGTTGCAGA GAGTTCATAG CTTCTAATGA TGAGAGAGAA AACTCTGCAG

421' TCATGTGCGC CGCTTGCGGC TGTCACCGGA GCTTCCACCG CAGGGTGGAG GAAGCCAGGG CTGGTTGATG
     ********** ********** ********** ********** ********** ********** * * **  **
421" TCATGTGCGC CGCTTGCGGC TGTCACCGGA GCTTCCACCG CAGGGTGGAG GAAGCCAGGG CAGTTTC-TG

491' ATATAGGGTA AAAAAAAAGC ATTATGATGA TGATGATGAT TGGGCATATA TATATATATA TATATATATA
     **  *               *  *  *  ***    *  * *        * *  *   **  **  *****   *
490" ATTAACACC CTTCCTTTTC TTCTTCTTGA ATCTCTTTAC CTTTTTTTTT TCCTTAATTA AATATAGCGA

561' TATATATATA TATATATA -----Undetermined----
     *   *      *    *
559" TTCTTTACGG TGCTCAAT GATTATCAAAACTTATATAGTTTTGATCTGAATGGATAAGATGTTGATATATATAT
     ATATATATATATGTATATATATTATCTCCCTTGTTTTGACAAGTGTCCATTCAGGTTGATGAATCAGTGAGCACCG
     CTCTCGTTTTGACAAGTGTCCATTTAGTTGATGATGAGTGAGCACTGTAAAATTTGAGGCATATATATATATATAT
     ATATATATATATATGAGTGTGTTGTTGTAATGTCTATGTAAGTTTGATGCATGACAGTTAATTTATATGCAGGCTG
     GTTGATGATATAGGGTAAAAAAAAAGCATTATGATGATGATGATGATTGGGCATATATATATATATATATATATAT
     ATATATATATATATATGTGGTGGAATCCATGGTG
```

Figure S5. Comparison of *GtMIF1-1* and *GtMIF1-2* sequences.

Start codon and stop codon are shown in red and green, respectively. Purple fonts are the sequence from the draft genome. Primers for *GtMIF1* marker are shown in blue font.

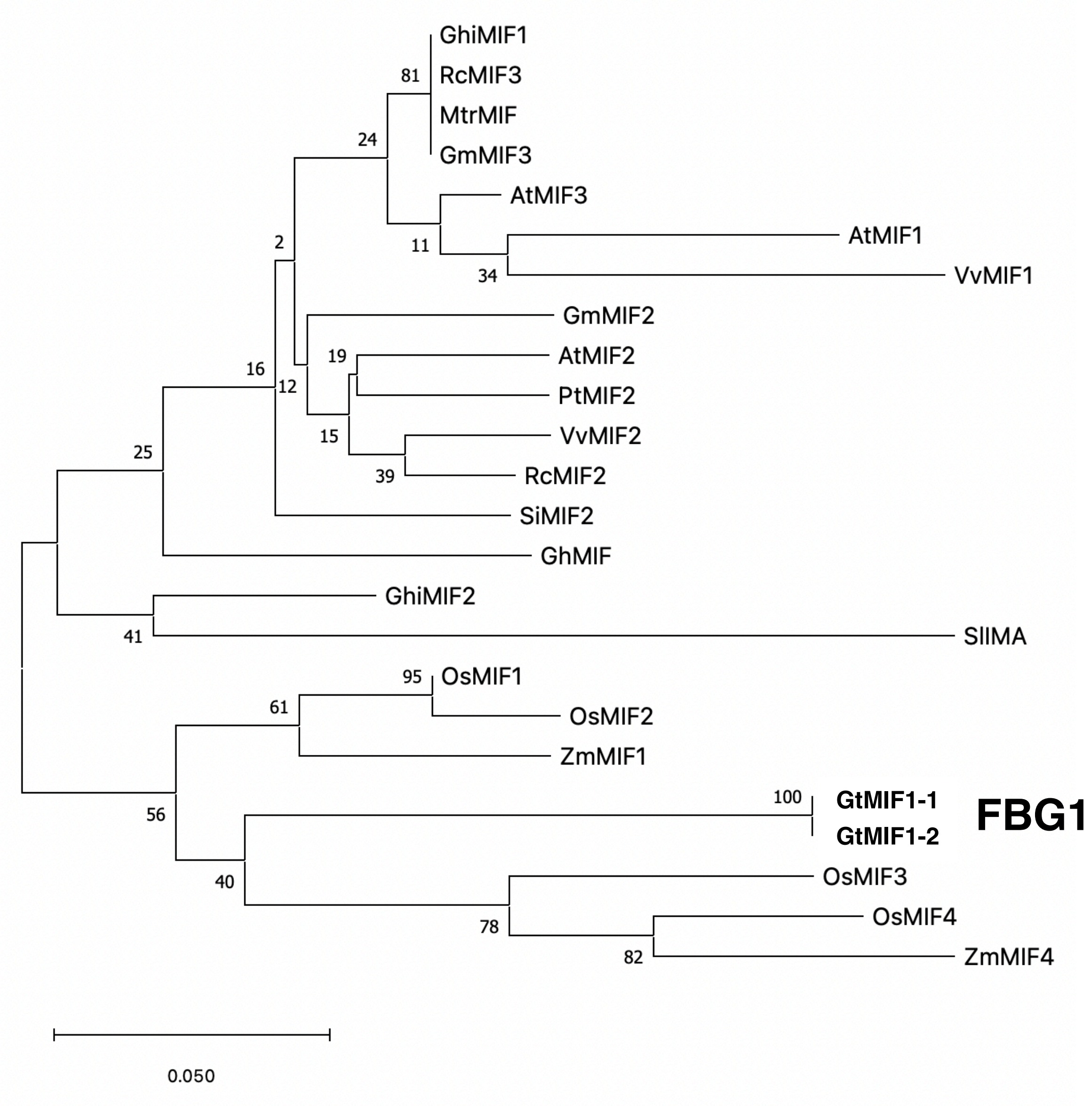

Figure S6. Neighbour-joining (NJ) phylogenetic tree of MINI ZINC-FINGER protein with GtMIF1-1 and GtMIF1-2.

The evolutionary history was inferred using the Neighbor-Joining method [1]. The optimal tree with the sum of branch length = 1.22361010 is shown. The percentage of replicate trees in which the associated taxa clustered together in the bootstrap test (1000 replicates) are shown next to the branches [2]. The tree is drawn to scale, with branch lengths in the same units as those of the evolutionary distances used to infer the phylogenetic tree. The evolutionary distances were computed using the p-distance method [3] and are in the units of the number of amino acid differences per site. This analysis involved 24 amino acid sequences. All positions containing gaps and missing data were eliminated (complete deletion option). There were a total of 43 positions in the final dataset. Evolutionary analyses were conducted in MEGA X [4][5]

1. Saitou N. and Nei M. (**1987**). The neighbor-joining method: A new method for reconstructing phylogenetic trees. *Molecular Biology and Evolution* **4**:406-425.
2. Felsenstein J. (**1985**). Confidence limits on phylogenies: An approach using the bootstrap. *Evolution* **39**:783-791.
3. Nei M. and Kumar S. (**2000**). *Molecular Evolution and Phylogenetics*. Oxford University Press, New York.
4. Kumar S., Stecher G., Li M., Knyaz C., and Tamura K. (**2018**). MEGA X: Molecular Evolutionary Genetics Analysis across computing platforms. *Molecular Biology and Evolution* **35**:1547-1549.
5. Stecher G., Tamura K., and Kumar S. (**2020**). Molecular Evolutionary Genetics Analysis (MEGA) for macOS. *Molecular Biology and Evolution* (https://doi.org/10.1093/molbev/msz312).

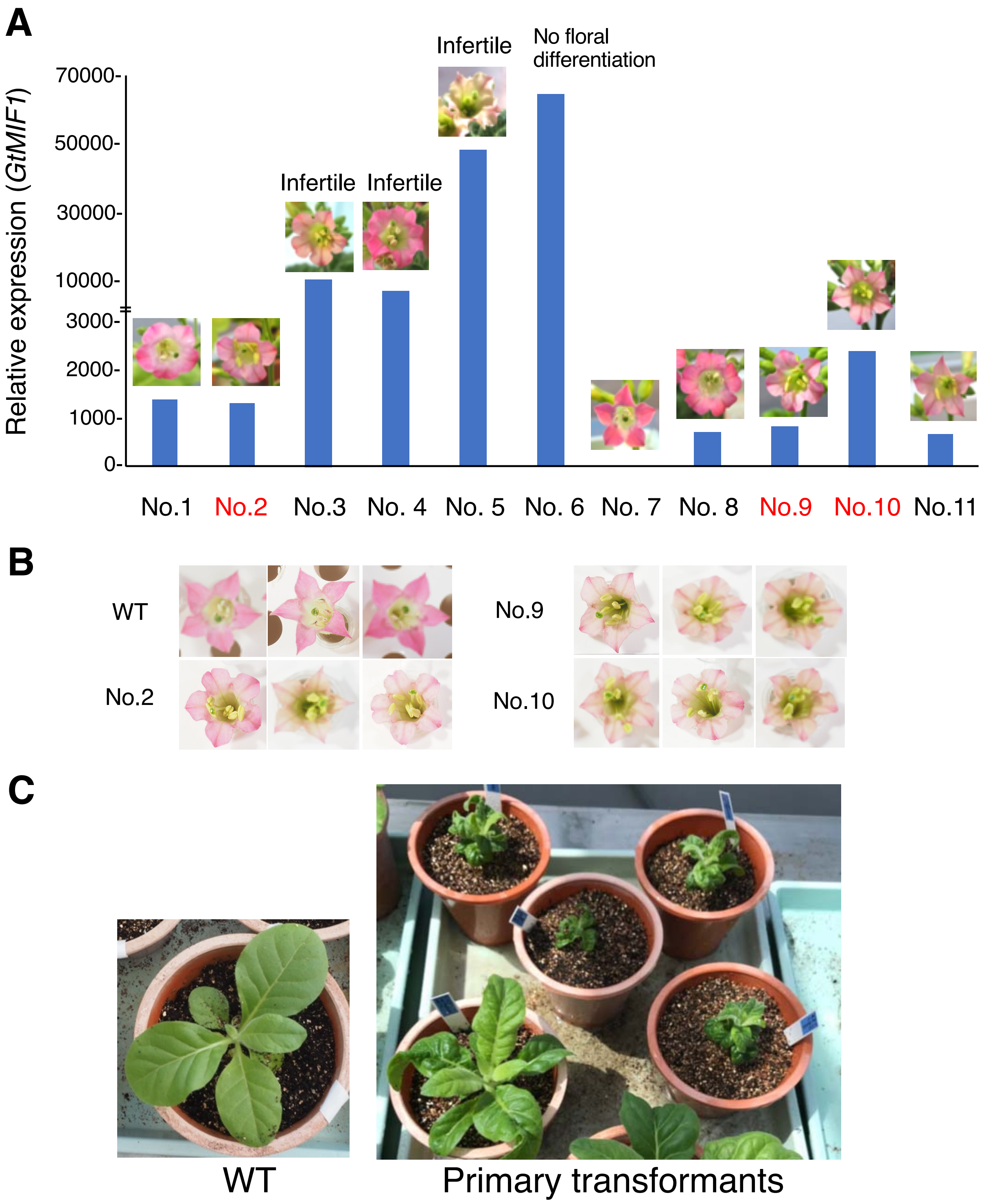

Figure S7. Phenotypes of *GtMIF1*-overexpressing tobacco (T_0).

(A) qRT-PCR analysis in leaves and typical flower photographs.

(B) Flower phenotypes of WT and selected three T_2 lines.

(C) WT untransformed control and primary transgenic plants showing severe phenotype.

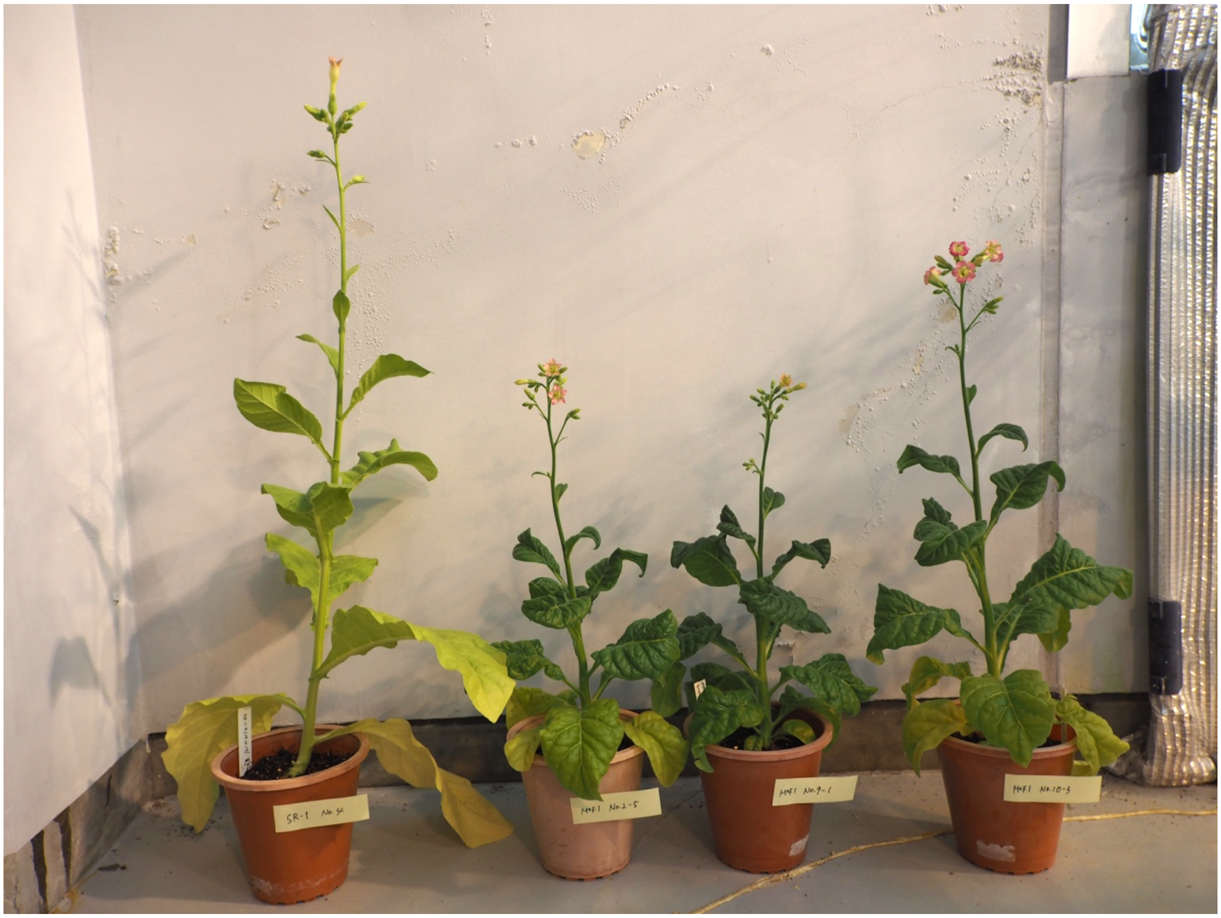

WT No.2 No.9 No.10

Figure S8. Plants at the flowering stage of an untransformed control (wild-type; WT) and *GtMIF1*-overexpressing tobacco T_1 lines.

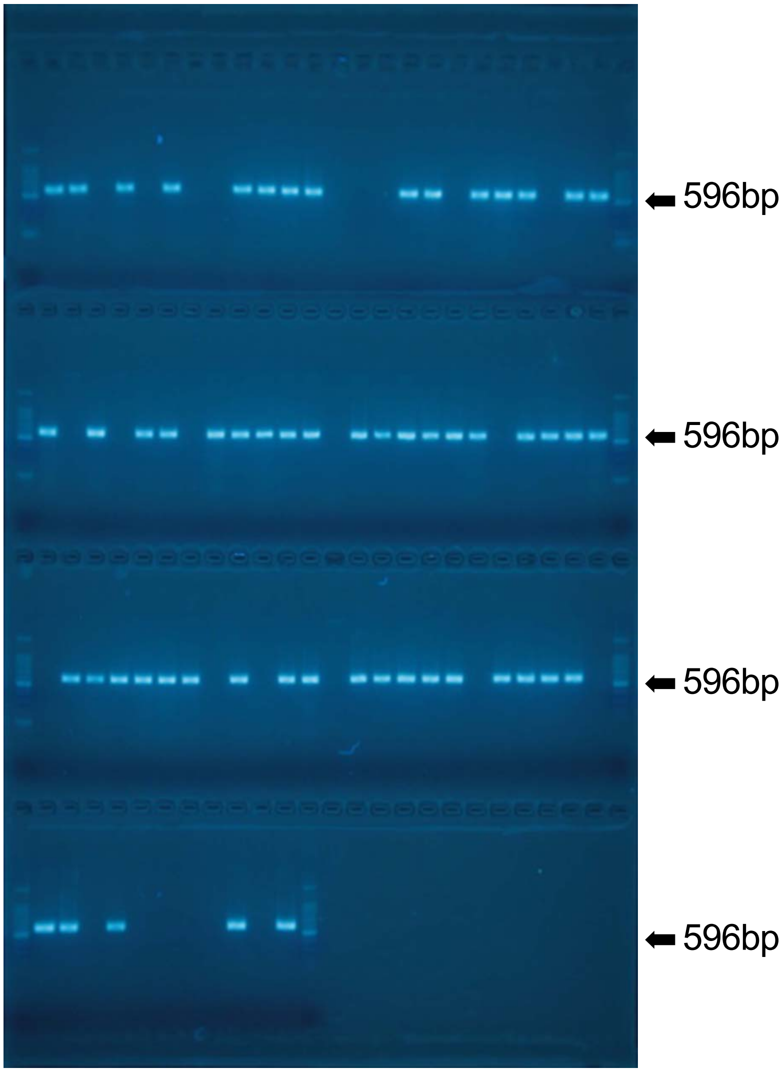

Figure S9. PCR analysis of the *GtMIF1* allele associated with faint-blue flower line in 83 F_2 progeny.

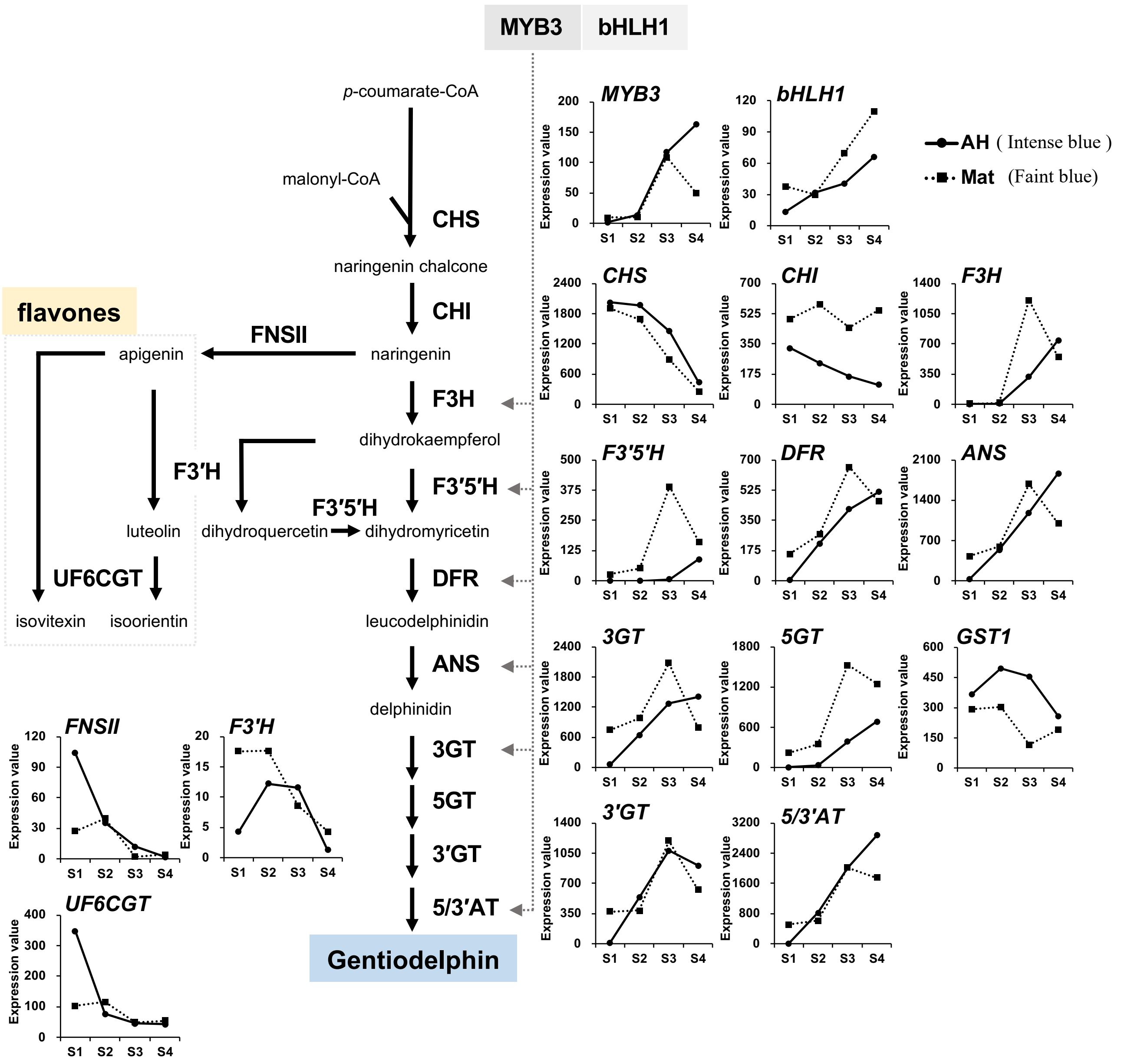

Figure S10. Expression patterns of genes in the flavonoid biosynthetic pathway in gentian flowers at four stages of flower development.

AH, 'Ashiro-no-Hatsuaki' -derived DH line (AH); Mat, Matsuo-derived DH line. Expression values are calculated by TMM normalization from FPKM values. Arrows indicate the catalytic reaction. Dotted arrows indicate the deduced transcriptional regulation based on our previous study (1, 2).

1. Nakatsuka, T., Haruta, K. S., Pitaksutheepong, C., Abe, Y., Kakizaki, Y., Yamamoto, K., Shimada, N., Yamamura, S., and Nishihara, M. (2008). Identification and characterization of R2R3-MYB and bHLH transcription factors regulating anthocyanin biosynthesis in gentian flowers. Plant & Cell Physiology ***49*** **:**1818-1829.

2. Nakatsuka, T., Saito, M., Yamada, E., Fujita, K., Kakizaki, Y., and Nishihara, M. (2012). Isolation and characterization of GtMYBP3 and GtMYBP4, orthologues of R2R3-MYB transcription factors that regulate early flavonoid biosynthesis, in gentian flowers. Journal of Experimental Botany ***63****:* 6505-6517.

Predicted protein(s):

>FGENESH: 1   1 exon (s) 923 -   1600   225 aa, chain +
MVTFRGNIPFNQDLAMLKSVNSTDPYIKLKQINQLYRKKNAQNQLNIPACEKSRSAVIDV
IILIAVVSACGVLLYPYMKLLVQTSADFFEEAVYVFKDEIDQAPLVYGCFGLSVLFALSA
LMAITLYTSRTCGKPTCRGLRKAAEFDIQLETEECVKSTTGVLGAGTKNGLVQLPRDHHR
ELEAELKKMAPPNGRAVLVFRARCGCPLGRLEVPGPKKMGRKFKK

>FGENESH: 2   4 exon (s) 2371 -   4194   100 aa, chain +
MKTSLPTPHRNCLPETVPWPVGPDTRLKAASSVYLCVLLVTPLTSSLGDPLLSRSSVCSS
FRPVIQYQTRKNMMYSLRSDLSKSVAKITHIKESVCAAVD

>FGENESH: 3   1 exon (s) 7463 -   7708   81 aa, chain -
MKKLHLVAKRESSSSSSSIITVRYMECQKNHAASIGGHVVDGCREFIASNDERENSAVMC
AACGCHRSFHRRVEEARAVSD

>FGENESH: 4   4 exon (s) 9656 - 11568   97 aa, chain +
MGPRSPTVQVERCKGRVLNEYGSNGSYQHGIRTGGTDGSTYGRGGRASAAGSINSSSQKY
SYMYIWFHVLSDSDRFLCFIGLGSGHFLCWTLTRAYP

>FGENESH: 5   1 exon (s) 13592 - 13873   93 aa, chain -
MIKKENVDRNEKERKCGQNRLGNIAFYCLQCLHVLCFFLLITITMHHGRRKINTTTSVPR
THSPVGKGRGRVKQPTERLEVRFPQGSNYSPPA

>FGENESH: 6   3 exon (s) 17022 - 18337   323 aa, chain -
MGRPPCCEKGGVKKGPWTPEEDIVLVSYLQEHGPGNWRAVPTNTGLRRCSKSCRLRWTNY
LRPGIRRGNFTDQEEKLIIQLQALLGNKWAAIASYLPERTDNDIKNYWNTHLKKKLKKMQ
QTGSDCSNSDELMSSHSLSKGQWERRLQADIKTAKQALQDALSLDKTIPQTPDVHPIHGY
YSFTKPVQTSTYASSTENIAKLLKGWTKSSPNSRSLSTQQSFNNAAAAADSTFSSEGSSV
ESKSGHDISEPFESNKFGFGSFDYSSSEFSQSIISSNERECKKENTADVPLSILENWLLE
DGGAPVKDELANDFLYYEQANLF

Figure S11. Prediction of potential genes in *GtMIF1*-containing contig.

The gene coding regions were predicted with FGENESH (http://linux1.softberry.com).
Sequence similarity search was performed by NCBI's blastp program.

FGENESH1: Ribosomal protein L34e superfamily protein
FGENESH2: Hypothetical protein. No hit to Arabidopsis protein database.
FGENESH3: GtMIF1 (FBG1)
FGENESH4: no hit
FGENESH5: no hit
FGENESH6: GtMYBP9 (R2R3-MYB protein of *Gentiana triflora*, accession no. AB733619)

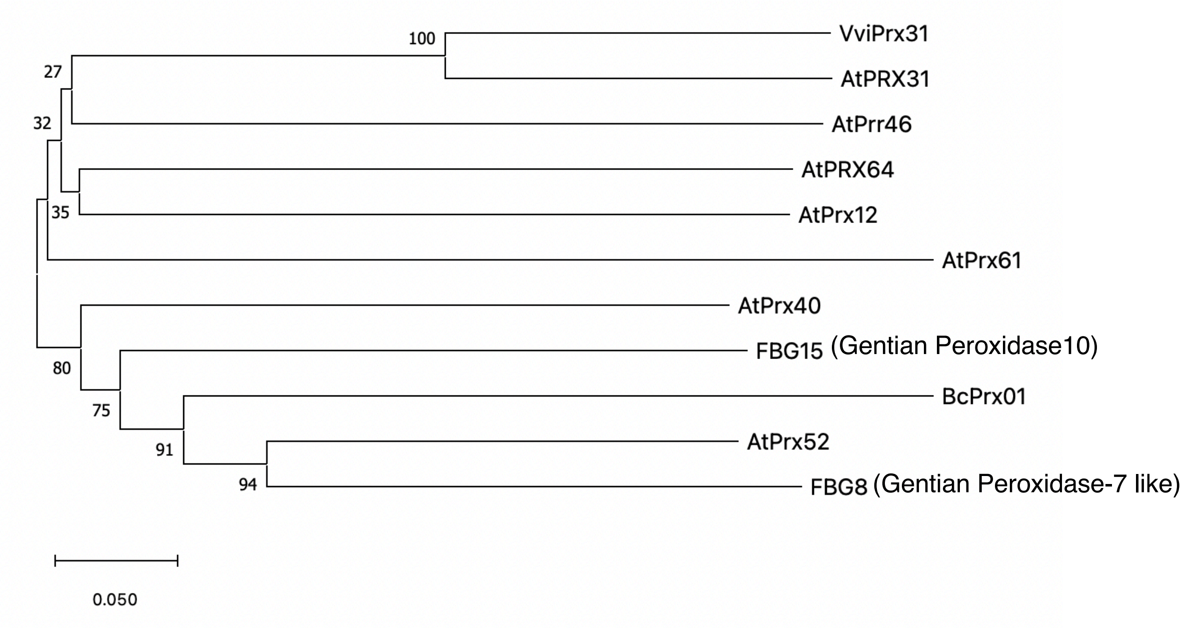

Figure S12. Phylogenetic analysis of FBG8 and FBG10 with several peroxidases.

The evolutionary history was inferred using the Neighbor-Joining method [1]. The optimal tree with the sum of branch length = 3.07162844 is shown. The percentage of replicate trees in which the associated taxa clustered together in the bootstrap test (1000 replicates) are shown next to the branches [2]. The tree is drawn to scale, with branch lengths in the same units as those of the evolutionary distances used to infer the phylogenetic tree. The evolutionary distances were computed using the p-distance method [3] and are in the units of the number of amino acid differences per site. This analysis involved 11 amino acid sequences. All positions containing gaps and missing data were eliminated (complete deletion option). There were a total of 282 positions in the final dataset. Evolutionary analyses were conducted in MEGA X [4][5]

1. Saitou N. and Nei M. (1987). The neighbor-joining method: A new method for reconstructing phylogenetic trees. Molecular Biology and Evolution 4:406-425.
2. Felsenstein J. (1985). Confidence limits on phylogenies: An approach using the bootstrap. Evolution 39:783-791.
3. Nei M. and Kumar S. (2000). Molecular Evolution and Phylogenetics. Oxford University Press, New York.
4. Kumar S., Stecher G., Li M., Knyaz C., and Tamura K. (2018). MEGA X: Molecular Evolutionary Genetics Analysis across computing platforms. Molecular Biology and Evolution 35:1547-1549.
5. Stecher G., Tamura K., and Kumar S. (2020). Molecular Evolutionary Genetics Analysis (MEGA) for macOS. Molecular Biology and Evolution (https://doi.org/10.1093/molbev/msz312). [4][5]
